# Understanding decision-making strategies in discrete choice experiment tasks when valuing health states that include duration, a cognitive interview study with Australian adults

**DOI:** 10.1007/s11136-026-04189-w

**Published:** 2026-03-04

**Authors:** Tessa Peasgood, Jill Carlton, Richard Norman, Donna Rowen, Marcel Jonker

**Affiliations:** 1 School of Medicine and Population Health, University of Sheffield, Sheffield, UK; 2 Curtin School of Population Health, Perth, Australia; 3 Erasmus School of Health Policy & Management, Erasmus University Rotterdam, Rotterdam, The Netherlands

**Keywords:** Qualitative, Discrete choice experiment, DCE with duration, Health state valuation, EQ-5D-5L

## Abstract

**Objectives:**

This study examined how participants engage with Discrete Choice Experiments (DCE) with duration tasks for valuing health states, including time preferences, and consideration of interactions between health and duration attributes. It also examined how task layout, colour, and language, particularly how dying is described, influenced decision-making.

**Methods:**

Twenty-one adults undertook online cognitive interviews while completing DCE tasks. Tasks involved health and duration attributes, with varying durations (weeks to 20 years) and split triplet designs in which respondents first choose between health states with the same duration and then between one of those health states and either full health for a shorter duration or immediate death. Data were analysed using Framework Analysis with iterative coding and a final thematic framework.

**Results:**

Participants showed willingness to trade life-years for better health, evidence of Maximum Endurable Time, and high value placed on final weeks for closure and goodbyes. Some participants interacted health attributes with duration where duration differed, while others did not. No additional clear evidence of discounting future time emerged. Some participants had emotive reactions to specific phrasing, underscoring the impact of inconsistent wording between choices. Overlapping domains, particularly combined with the use of colour, made tasks easier but sometimes led to ignored domains when duration varied. Participants struggled with hypothetical scenarios and unfamiliar health attributes.

**Conclusions:**

Considering the complexity of decision-making and the influence of framing, presentation and language can inform design and modelling choices for DCE with duration studies.

**Supplementary Information:**

The online version contains supplementary material available at 10.1007/s11136-026-04189-w.

## Highlights

Little is known about individuals’ decision-making when answering DCE with duration questions, particularly whether they adopt a non-zero-time preference or consider interactions between the duration and attributes in a choice set.

In cognitive interviews with adults we found that choices were influenced by Maximal Endurable Time and the importance of the final weeks before death. Participants did not articulate other clear evidence of discounting future time. Language, colour, and holding attributes constant within choice sets can affect thought-processes and this should be considered during DCE design. While overlap safely simplifies choice tasks with equal durations, it can prevent multiplicative reasoning when durations differ.

**Supplementary Information:**

The online version contains supplementary material available at 10.1007/s11136-026-04189-w.

## Introduction

Discrete choice experiments (DCEs) are widely used to develop value sets for preference-weighted measures used in economic evaluation [1–4]. Their application relies on several assumptions about how respondents make choices between health states, including how health attributes and duration are processed, and how individuals trade quality of life against length of life. Increasingly, these assumptions have been questioned, raising concerns about whether commonly applied modelling frameworks adequately reflect respondents’ decision-making processes.

In DCE valuation studies that incorporate duration as a separate attribute (referred to here as ‘DCE with duration (DCEd) mixed approach’), data have typically been analysed using utility specifications designed to align with the Quality Adjusted Life Year (QALY) framework. These models generally assume that the value of time depends on the quality of the health state experienced, such that duration interacts with health-state levels within the utility function. This implies a multiplicative relationship between health domains and time and allows the latent DCE utility scale to be anchored at 1 for full health and 0 for dead (i.e. zero duration) [5, 6].

Whether respondents actually evaluate choices in this multiplicative way has been contested [7]. Such models require complex decision-making processing, as respondents must assess the value of duration conditional on the health state. In practice, respondents may simplify tasks by treating duration as an independent attribute, implying an additive utility function across health and time. Simplifying heuristics, such as attribute non-attendance [7–10], have been identified in DCEs, particularly when tasks are complex or involve many attributes [11], and are more common among older or less educated respondents [12, 13]. Consistent with this, analysis of EQ-5D-5L and SF-6D ‘DCEd mixed approach’ valuation data found that the data were best modelled by assuming that most respondents adopted an additive, rather than multiplicative, utility function [7].

Assumptions regarding how individuals value time in DCEs with duration have also been challenged. DCE-with-duration models have often assumed constant proportional trade-off, implying that respondents are willing to trade a constant proportion of remaining life years for a given health gain, regardless of the total duration involved. This assumption is consistent with zero discounting of future time. However, evidence from both time trade-off (TTO) [14] and DCEd [8, 15] data suggests that individuals exhibit non-linear time preferences. Positive time preference, whereby time in the more distant future is valued less than time closer to the present, has been observed, and allowing for such preferences in DCE models has been shown to align results more closely with those obtained using TTO methods [16].

In addition to a positive time preference, individuals’ willingness to trade quantity for quality of life may vary with duration if very poor health states are considered acceptable over short periods but unacceptable for long durations. This duration dependence can arise for at least two distinct but related reasons. Firstly, people may perceive very poor health states as tolerable for a short period but intolerable if lived in for a long period. This implies that the value of such states becomes negative beyond a certain duration, a phenomenon referred to as Maximal (or maximum) Endurable Time (MET) [17, 18]. Such preferences have been observed in both TTO and standard gamble (SG) [19]. Secondly, apparent preferences for a poor health state over immediate death may not reflect the intrinsic value of the health state itself, but rather an aversion to dying immediately. Such aversion may be driven by practical or emotional concerns, such as the desire to say goodbye to loved ones or prepare for death. In these cases, choices may reflect preferences over the timing or process of death rather than the value of the health state per se.

Several DCE formats have been developed to value health states while explicitly incorporating duration. A more recently developed format is the ‘DCEd split triplet design’ in which respondents first choose between two health states of equal duration, followed by a second choice between one of those states and either full health for a shorter duration or immediate death. The split triplet design aims to reduce task complexity by avoiding simultaneous comparisons of impaired health states with differing durations, thereby reducing reliance on assumptions about how respondents combine duration and health. However, as a relatively new method, little qualitative work has examined how respondents understand and engage with this design.

Despite the relevance of decision-making assumptions, time preferences, and death-related considerations for DCEd valuation models, their influence on decision-making has not been explored qualitatively. Qualitative methods such as think-aloud and cognitive interviewing are commonly used to improve survey design [20, 21]. Such methods have also been used to explore respondents thought-processes in DCE tasks for health prioritisation [13, 22, 23] and for health state valuation [24–30]. Although qualitative work to develop and refine attributes within DCEs is now standard, little attention has been paid to how respondents interpret differences in duration followed by death or the impact of language choice around the ‘dead’ state in the DCEd split triplet design, despite the potential for subtle language differences to impact upon respondent choices and their overall experience of engaging with the research. This is an important omission given the increasing use of ‘DCEd mixed approach’ and ‘DCEd split triplet design’ in health state valuation [3]. The initial DCEd split triplet designs incorporated colour-intensity shading using a colour-blind friendly purple palette [31, 32] and bolding of levels. This layout and colour scheme has been shown to be appropriate using quantitative assessment methods (e.g., higher choice consistency, lower survey drop out, and reduced attribute non-attendance), but little qualitative work has been undertaken to understand how these design choices may influence decision-making processes.

This study builds on previous qualitative research exploring participant decision-making and interpretations of different durations in DCE tasks, with a particular focus on how respondents engage with the new ‘DCEd split triplet design’ compared with other DCEd formats. The study had two main aims:

1. To examine how participants approach DCEd tasks, particularly choice sets within the ‘DCEd mixed approach’ and ‘DCEd split triplet design’, and what this reveals about how they interpret time and their preferences towards time in the future.

This aim includes exploring:

- whether participants show signs of adopting additive, multiplicative or some other approach in ‘DCEd mixed approach’ tasks;
- whether they view time in the distant future as less valuable than time in the near future (i.e. evidence of non-zero time preference);
- whether their decisions reflect considerations of ‘maximal endurable time’ for severe health states (i.e., judging certain health states as better than dead when lived in for short durations but worse than dead when lived in for long durations); and.
- whether participants apply a distinct value of time immediately before death, such as applying a discontinuous discount rate for very small durations of life.

2. To enhance understanding of how participants interact with and interpret standard DCE interfaces for valuing health states, including (a) layout, (b) colour and (c) language choices, particularly in relation to the description of the ‘dead’ state and of passing away.

## Methods

### Recruitment

Data saturation was expected after 20 interviews, based on existing literature [33]. A purposive sampling approach ensured a diverse mix of participants across gender, age, and education, as preferences can vary by age and gender [34]. Participants had to be 18 or older, live in Australia, and speak fluent English. Recruitment was conducted by Pureprofile, a panel company. Interested members completed a short screening survey covering gender, age (18–35, 36–50, 50 + years), and education level (no post-school qualifications, certificate/diploma, or bachelor’s degree/equivalent). Pureprofile selected participants to achieve the purposive sample, who were then invited to a one-hour Zoom interview. They received AUD$25 as compensation.

### Interview process

**Fig. 1:**
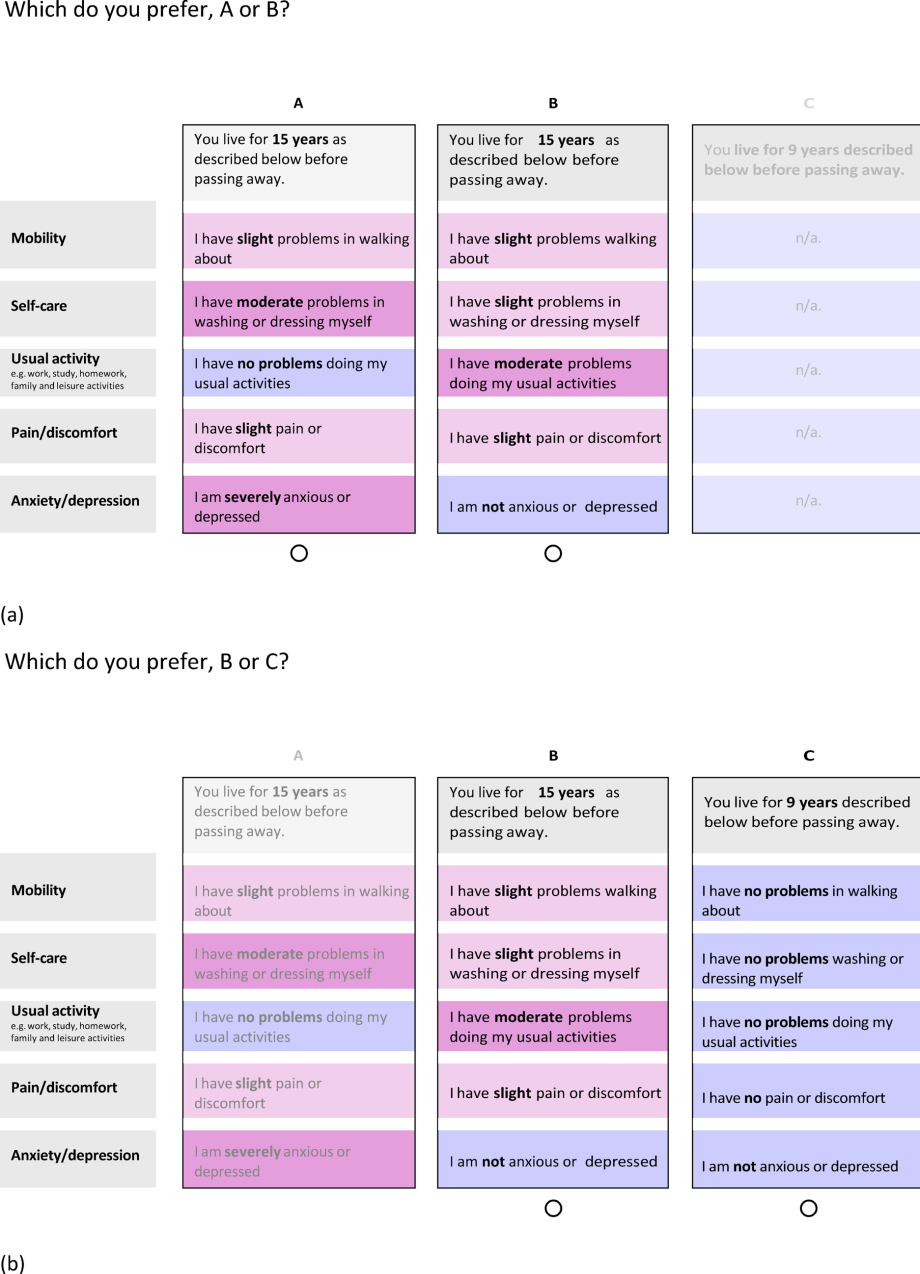
**a** Example split triplet DCE task 2, **b** Which do you prefer, B or C?

We conducted cognitive interviews using retrospective probing techniques [35] to explore how participants interpreted and completed DCE tasks. Two pilot interviews were conducted with a convenience sample to test and refine the interview prompts (see Supplementary Materials 1 (SM1)). Interviews were conducted by two experienced qualitative researchers (TP and JC); researcher positionality is discussed in SM2. At the start, researchers recapped the study aims, outlined the interview process, and obtained verbal consent. Survey questions were displayed on screen. Each interview had two parts. Part 1 collected sociodemographic information and participant’s self-reported health via EQ-5D-5 L (SM3). Part 2 focused on the choice tasks, including examples of ‘DCEd mixed approach’ and ‘DCEd split triplet designs’ with durations ranging from weeks to 20 years and health described using EQ-5D-5L. Short durations (weeks–months) were included to explore preferences for near-term outcomes, including the final weeks of life, while longer durations (years) were included to explore time preference. Exact duration pairs were iteratively adjusted during interviews based on participants’ responses to probe their point of indifference between health states and length of life, consistent with cognitive interviewing techniques. Example DCEd split triplet tasks are shown in Fig. 1a and b. The order of questions was kept broadly consistent, with more difficult or emotionally challenging questions toward the end.

The DCE tasks and associated interview probes were chosen to address each of the study aims and were largely consistent across interviews. Participants typically discussed around16 DCE tasks, supplemented by ad hoc verbal scenarios with alternative durations to allow for further probing and clarification of preferences. The general alignment of tasks and probes to study aims is shown in Table 1.

**Table 1:** Interview tasks, probes and study aims

	Questions	Probing topics	Aim (number)
Warm up	Paired choice question on fruit & two paired choice questions with duration held constant.	Participant understanding of the tasks	Put at ease Assess general understanding of tasks
Q1	Paired choice with duration held constant	General decision process Interpretation of colours	Assess interpretation of layout and colour (2a, 2b)
Q2	Paired choice with impaired health state for 10 years and full health for less than 10 years in split triplet design	General decision process Reflections on trading off time	Assess willingness to trade years of life and understand thought processes (2a)
Q3	Paired choice with some attributes held constant including duration	General decision process. Reflections on attributes that were held constant Language choice of ‘passing away’	Assess interpretation of layout and impact of holding attributes constant (2c)
Q4	Paired choice with duration varied	Explore the decision process with probing around how differences in duration were considered Explore whether the whole health state was considered as being experienced for the given number of years or whether attribute levels, including duration, were compared independently Considered how attributes that were held constant were treated. Where possible, use indirect probes e.g. ‘can you say a bit more about how you used the information to make your decision’ to avoid leading	Distinguish between multiplicative vs. additive approaches (1a)
Q5	Paired choice between 3 weeks in a mildly impaired health state and dying immediately	Probe preferences towards final weeks. Probe impact of language ‘immediately pass away (e.g. due to a heart attack)’	Understand the importance (or not) of the final weeks of life Understand potential impact language and choice of example (2c)
Q6	Multiple paired choices with duration varying between choices and choice sets varying in near/distant future, sometimes comparing choices with constant proportional time loss (e.g. 9 vs. 15, 18 vs. 30), others with different proportional time loss (e.g. 9 vs. 15, 18 vs. 25)	Probe preferences towards time in the future	Assess potential for impact of discounting future time gains (1b)
Q7	Paired choices between very poor health states with duration constant	Probe decision making	Assess views on states considered worse than dead
Q8	Paired choices with very poor health states with duration varying	Probe and ask additional tasks with more/less time in very poor health states Probe whether a longer time in the health state would be preferable	Assess presence of MET (1c, 1d)
Q9	Paired choice with very poor health state vs. immediate death, compared to vs. 1 week in full health	Probe decision making	Assess presence of MET Reflect on impact of offering 1 week of time prior to death (1c, 1d)
Q10	Paired choice with very poor health states	Probe any differences in ability to undertake tasks when states are all very poor	Assess any differences in decision making processes when states are very severe compared to when they are mild
Q11& Q12	Paired choice: 1 year in poor health vs. 6 months in poor health and 6 months in poor health vs. immediately pass away Depending upon responses alternative durations compared	Probe decision making for participants who select less time in the state in the first choice but prefer to live for 6 months in the second choice	Assess presence of MET (1c)
Q13	Paired choice: 8 years very poor health vs. 1 week in good health.	Probe whether 1 week makes a difference to the choice when compared with dying immediate	Assess distinct value of final days/weeks (1d)
Q14 & Q15	Paired comparison between worse (pits) state for different time periods (e.g. 3 years vs. 3 months)	Where the participant prefers a shorter time in the worst health state ask whether living for the shorter time in the health state is preferred to immediate death	Assess presence of MET (1c)

KEY: * Study aims are to consider whether participants adopt additive, multiplicative or some other approach in ‘DCEd mixed approach’ tasks (1a), adopt a non-zero time preference (1b), express considerations of ‘maximal endurable time’ for severe health states (1c), adopt a distinct value of time immediately before death (1d), and to consider participants interpretation of layout (2a), colour (2b) and language choices particularly to describe dying (2c)

Throughout Part 2, participants were encouraged to describe their interpretation of each task. Retrospective probes explored understanding, reasoning and difficulties (see SM3 for the topic guide and example survey questions). Interviews were audio-recorded and transcripts were generated using Zoom and subsequently verified for accuracy by the interviewer. Reflective notes were taken during and after each interview to support analysis. Ethical approval was granted by The University of Melbourne Human Research Ethics Committee (ID 2057011.1) with further considerations detailed in SM4.

### Data analysis method

An iterative analytical approach was adopted, with analysis conducted alongside data collection [36]. This allowed refinement of the topic guide to explore emerging themes. Data collection and analysis continued until saturation was reached in a sample sufficiently broad across the pre-agreed sampling characteristics of age and gender.

Transcripts were analysed using Framework Analysis [37] following Gale et al. [38], which allows systematic coding and categorisation which maintaining a clear link to original data. Researchers familiarised themselves with the data by listening to the interviews, transcribing and reading interview notes. Next, the first two transcripts were independently coded by two researchers using a ‘top-down’ approach based on study aims while noting emerging ‘bottom-up’ themes. The framework was discussed and modified accordingly. Some initial codes which did not pertain directly to the main study aims were later dropped. Codes were organised within superordinate ‘categories’ where appropriate, with initial descriptive summaries for each code. The revised framework was subsequently applied to two additional transcripts, both of which were double-coded. Further refinements were made to the framework based on this process. An additional transcript was then double-coded and discussed to ensure consistency and rigour, resulting in consensus on the final framework. The finalised framework was thereafter applied to all the complete set of transcripts. Excel was used to organise excerpts and synthesise codes and patterns.

## Results

### Sample

Twenty-one participants were interviewed online between 25th May and 25th June 2021; interviews lasted 27 and 56 minutes, see SM6 for participant characteristics.

### Overview of themes

Analysis identified three overarching themes: ‘factors influencing decision-making’, ‘process of decision-making’ and ‘influence of survey design’. The theme and sub-theme codes can be seen in SM7.

Participants generally engaged carefully with the tasks. Decision-making varied in willingness to trade length of life for quality, with many participants demonstrating MET for poor health states and placing particular importance on time near death. Participants differed in whether they integrated duration and health multiplicatively or treated them as separate considerations. Survey design, including the wording used to describe death and colour-shading, shaped participants’ understanding and sometimes led to simplifying heuristics, errors, or inconsistent choices. Findings are presented below by theme and sub-theme.

### Factors influencing decision-making

#### Willingness to trade life years

Participants varied in willingness to give up years of life in favour of improved quality of life although all traded in at least some tasks. Reasons included wanting time with loved ones (Table SM8 Quotes(Q) Q1-4), hoping for medical breakthroughs (Q5-7), beliefs about life’s inherent value (Q8-9), prioritising quality over length of life (Q10-11), concern about being a burden (Q12-14), or judging states worse than death (Q15-18).

#### Maximal endurable time

Almost half of participants made choices and/or described considerations that demonstrated MET (Q19-26) in which they preferred a given health state over immediate death whilst also preferring a shorter rather than longer time in that state. Participants describe how they could “*hack it out*” (Q19), “*soldier on*” (Q20), “*bear with this – just for that short period of time*” (Q21), but beyond a certain duration they selected the shorter duration so they “*would not suffer as much*” (Q22). The optimum duration or “*tipping point*” (Q23) of a very poor health state ranged from 3 months to 2 years (Q24-24).

#### Value of time in the final weeks

Participants considered ‘living 1 week’ (or ‘3 weeks’) versus ‘dying immediately’. Some felt the difference was unimportant (Q28-29), but most noted the importance of final few weeks to allow time for preparing for death (e.g., saying goodbyes, getting affairs in order, providing computer passwords, and having a “*level of closure*”) (Q30-34). One considered the impact of dying immediately on others, including missing final farewells (Q34).

#### Value of time in the near and Far future

The interview presented choice tasks with various differences in durations, some of which occurred in the near time (e.g., 3 months, 1 year, 2 years) and some occurred further into the future (e.g., 10, 15, 18 years) to explore how people thought about trade-offs at different times in the future and subsequently infer whether participants’ appeared to discount future gains. Some participants gave less weight to duration differences further into the future based on the age they would be at that point in time, reasoning that they would be elderly and likely have additional health problems (Q35-36). This view mirrored that of some participants who noted that if older people were asked these questions, they would likely give less priority to additional years of life (Q37-39).

Some participants did not give much weight to the difference between 8 and 10 years or between 15 and 18 years (Q40-45) seeing 2 or 3 years difference at that point in the far future as “*insignificant*” (Q42) and the durations as *“virtually the same*” (Q41). Differences in duration between the choice set that occurred into the far future were treated differently by some participants, but others treated duration differences in absolute terms and as the same value (e.g. when comparing [15 vs 18] against [2 vs. 5] one participant noted “*the difference is still the same*” (Q46). Two participants articulated judgements consistent with a consideration of constant proportional trade off (Q47-48).

### Process of decision-making

#### Interpreting the health state and choice offered

When answering the choice questions, participants drew upon their experiences of health (personal or that of family, friends and acquaintances) (Q49-52). Some noted that a lack of experience in some health attributes made the choice hard (Q53-58). Participants reported some health states to be unrealistic (Q59-60), which made their decision-making difficult (Q61-62). Participants reported uncertainty due to other factors including whether pain medication/other treatments were available (Q63-65), and lack of clarity of what ‘usual activities’ incorporated (Q66). Participants noted the uncertainty inherent in making hypothetical choices and recognised that their choices may differ if they faced the situation in real life (Q67-68). Some participants reflected upon how the severity of depression would influence the impact on quality of life of another health attribute based on ability to cope with, and receive support for, other problems (Q69-70).

#### Multiplicative or additive thought process

The interviewers probed the decision-making process for choices with different durations with the aim of understanding whether the participant adopted a multiplicative approach or an additive approach. Some participants described a multiplicative approach, for example by demonstrating that they considered the duration difference interacting with the health state (Q71-73). However, others appeared to separate out the two considerations (Q74). In choice sets in which duration differed but some health attributes had the same severity, some participants ignored those attributes describing that they “*just put a line through it*” (Q75) or they “*basically eliminate them if they are the same*” (Q76). This suggests that for these participants health attributes that do not vary within a choice set are not considered in a manner in which they interact with the duration.

### Influence of survey design

#### Wording to describe the ‘dead state’

Participants were asked about terms used to denote death. The DCE choices with duration stated, ‘You live for ‘x’ years described below before passing away’ and the interviewer raised other possible descriptors (e.g., ‘dying’, ‘dying immediately’, ‘passing away peacefully in your sleep’, ‘being dead’ and ‘not living any time at all’) to see if they were interpreted differently or preferred. Views were mixed. Some preferred the softer, kinder, more polite tone of the term ‘pass away’ over ‘dying’ (Q77-82). For some, ‘pass away’ and ‘dying’ implied different meanings (Q83-84), while others had no preference (Q85) or favoured ‘dying’ (Q86). One noted that ‘dying’ was clearer, especially for non-native English speakers, as euphemisms could be confusing (Q87).

The term ‘dying immediately’ evoked an emotional response for some (Q88-89), whilst the phrase ‘not living any time at all’ was generally disliked (Q90-93). One participant suggested ‘before your life ends’ as a more neutral alternative (Q94). Perception of ‘dying immediately’ varied - some saw it as sudden death (Q95-97), others as a transition into palliative care (Q98-99).

**Fig. 2:**
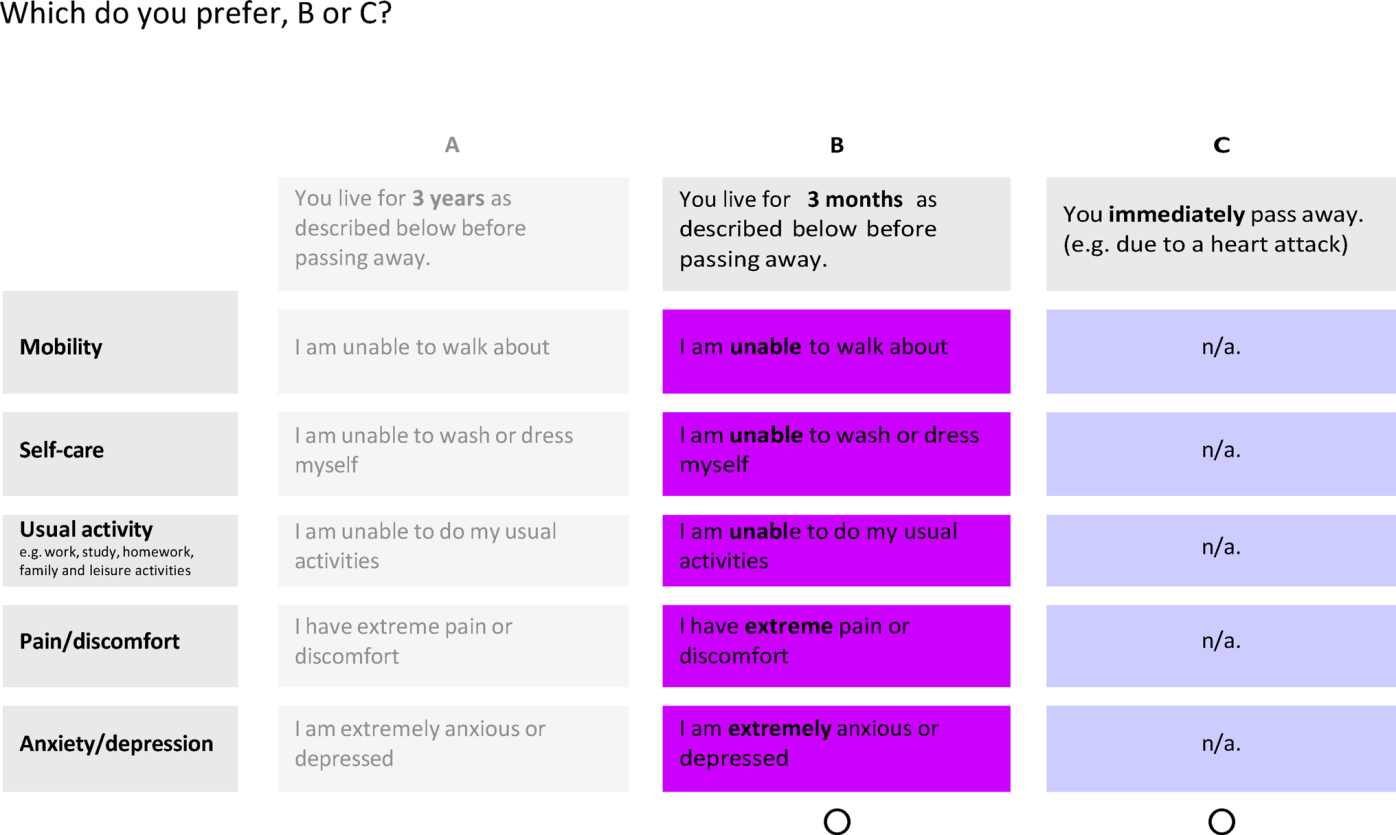
Example DCE question with a choice of ‘immediately pass away’

The DCE choice option of immediate death was worded as ‘You immediately pass away (e.g., due to a heart attack)’, as this was the description being considered for ongoing research using the ‘DCEd split triplet design’ approach. Interviews explored participants’ views of this wording and the example provided (i.e., heart attack) (see example in Fig. 2).

Many participants found the heart attack example to be appropriate, noting that it was clear and realistic (Q100-104), though some described it as “*scary*” or “*harsh*” (Q105-106). Some inferred added meaning, thinking that this option might be harder on loved ones but offer an appealingly quick death for themselves. Others interpreted the option without the example (i.e., ‘You live for X years as described below before passing away’) differently (Q107-109). Some thought about a better death than a heart attack (Q111), others thought the alternative unspecified cause of death was more threatening because its absence implied something worse than a heart attack (Q112). Participants suggested using consistent wording in both options (Q110-112).

The phrase ‘pass away peacefully in your sleep’ was seen as less realistic than a heart attack (Q113-116), especially for younger people (Q115-116). One participant thought the heart attack example could be problematic or triggering for someone with recent experience of a heart attack (Q117).

#### Use of colour-shading and bolding of text

Colour and shading were used to emphasise attribute severity, from light pink (mild) to dark purple (severe), with the severity level also shown in bold text. Participants found the design appealing (Q118) and said that colours and bolding helped identify severity levels and matching attributes within a choice set (Q119-126). Some relied heavily upon the colours, sometimes ignoring the attributes of the same colour even when duration varied (e.g., *“I don’t need to read that”*) or using a colour counting heuristic (Q137-129). However, not all participants found colours useful - two were indifferent (Q31-32), while others were unclear on their meaning, didn’t notice them or found them distracting (Q132-133).

#### Potential errors and inconsistencies

Several errors and inconsistencies were observed during interviews. Some participants overlooked differences in years within at least one choice task. They explained that this may have been due to the lack of colour in that section or the absence of a duration heading (Q134-136). Two initially selected the option closest to their own health (Q137-138). Some tried to select the greyed-out option of the DCEd split triplet choices and were confused by three visible choices (Q139). One did not understand the dead state or its lack of a time frame (Q140). Another re-interpreted the severity of the state mid-task changing their description of an attribute from ‘moderate’ to ‘very’ (Q141). Some assumed that some aspects of the health state could be altered through intervention(s) (i.e., including medication/therapy for depression, and treatment for pain) (Q142-144).

One participant, who the interviewer judged as engaged and thoughtful, gave inconsistent choices, prioritising a longer duration in poor health when thinking about family but preferring immediate death when focusing on perceived cleanness of a heart attack (Q145, showing 3 years in 55555 > immediate death, and immediate death > 6 months in 15543).

## Discussion

This study used cognitive interviews with retrospective probing to better understand the decision-making processes which occur during DCEd and DCEd split triplet tasks. We found that participants had minimal difficulties completing the tasks, although they did not always complete them as intended. As demonstrated in similar studies [25, 26], participants drew upon personal experiences when thinking about health attributes, expressed uncertainty in interpreting less familiar attributes and inferred additional information (such as the state changing due to taking pain medication). We identified two participants who selected the option that was closest to their own health - as also observed in think-aloud DCE studies with people with dementia and their carers [27] and with young people [39]. Such errors may not be picked up in unsupported self-complete surveys or through standard quality control checks.

The use of colour was perceived to make the task easier, however, we identified cases of relying heavily on colours. This may lead respondents to pay less attention to the domains and respond at speed.

We identified inconsistencies within the choices of participants who were fully engaged with the tasks, but thinking about different considerations at different points throughout the interview. This within-person variation has been identified in DCE studies as ‘learning effects’ in which the act of completing the DCE constructs individual preferences [40]. Researchers should be cautious when applying strict exclusions based on inconsistent responses, which may exclude considered responses.

Participants brought additional considerations to the description of the state around the mode of death. The idea of an immediate death was emotive and unpalatable for some. In reflecting on the impact of the wording and description of dying, participants noted language around death should be constant between choices to avoid this influencing their decision.

The study sought to explore four specific aspects of participant’s time preference. Firstly, whether participants had a non-zero-time preference. Whilst specific choice questions and interview prompts were designed to encourage discussion on this element, it was sometimes difficult to identify participants’ use of non-zero-time preference and distinguish this from adopting constant proportional trade off. Some participants did consider future uncertainty, but this mostly related to the health state and whether additional problems would be likely due to them having aged or experiencing less problems due to new medical breakthroughs. Some participants expressed that they gave very little weight to duration differences in the future, however, they found it hard to articulate whether time in the far future was being considered as less valuable.

Secondly, to explore whether participants’ preferences aligned with the concept of MET when considering severe health states. We found strong evidence of this, with almost half of all participants expressing MET preferences; with participants suggesting various optimum times for living in very poor health states from 3 months to two years. Given this, how MET impacts upon preferences should be a consideration in the choice of elicitation method.

Thirdly, to explore any distinct value of time immediately prior to death, and identify any indications that participants adopted a discontinuous time preference for very small durations of life. We found some evidence of participants doing so. Participants described the importance of having time to say goodbyes and put affairs in order, alongside an emotive reaction to the idea of immediate death which they interpreted as having negative consequences on loved ones. This may imply an additional reluctance to select the immediate death option beyond respondent’s judgement of the utility of the two states.

Finally, to identify whether participants adopt an additive or multiplicative decision-making approach when answering DCE questions in which duration varied. We found evidence of both approaches. Fixing health attributes across profiles within a choice set (particularly when paired with colour to show the severity) led several participants to consciously ignore the constant attributes hence these attributes are not interacted with duration. This phenomenon has been documented by others in DCEd; Ratcliffe et al. [27] observed participants removing attributes with the same levels from their consideration. That some participants adopt an additive approach is an important limitation for the ‘DCE with duration (DCEd) mixed approach’ which models the data based on an assumption of multiplicative decision-making.

This study has several notable strengths. The use of cognitive interviews with retrospective probing generated rich insights into how participants interpreted and completed both the DCEd mixed approach and the split triplet design. Furthermore, the rigorous methodological approach (e.g., iterative data collection and analysis, dual coding, and consensus building) enhances the validity of the study’s findings.

The study was not without its limitations. The findings were not returned to participants for verification, which may have limited opportunities to confirm the interpretation of the results. Recruitment through a commercial panel can include participants who are not representative in terms of interview experience and/or motivation. Furthermore, the act of thinking aloud and responding to prompts may alter participant’s level of concentration and thought processes [41] compared to independent, and likely much faster, completion. The ‘think-aloud’ and retrospective cognitive probing approach was not able to clearly evidence the use of time discounting, this may be because the effect was too small or even sub-conscious and hence not easily explored in qualitative interviews. Finally, it is possible that the order in which topics were discussed in the interviews influenced the way in which participants responded. Randomisation of tasks (i.e., choice sets) was not used due to ethical concerns related to discussion of sensitive topics.

## Conclusion

Participants’ interpretations of future time when completing DCEd tasks is complex. The final weeks of life held particular importance for some, and very poor health states were subject to MET for nearly half of participants. Some participants disregarded differences in duration occurring in the far future. Thought processes can be influenced by small changes in wording in the choice sets, particularly around the description of dying, and from design choices, such as the use of colour and overlap which encouraged participants to ignore attributes that are the same even where duration varied. The complexity of decision-making and framing effects in presentation, language and trade-offs need to be considered when designing and modelling DCE with duration studies. We recommend extra caution of using level overlap in a ‘DCEd mixed approach’ if data is to be analysed assuming a multiplicative approach.

## Supplementary Information

Below is the link to the electronic supplementary material.

Supplementary Material 1
