# Supplementary material for: Understanding decision-making strategies in discrete choice experiment tasks when valuing health states that include duration, a cognitive interview study with Australian adults
Source: Qual Life Res. 2026 Mar 4;35(4):91. doi: 10.1007/s11136-026-04189-w (PMC12960390; doi:10.1007/s11136-026-04189-w)
Supplement: Supplementary file 1 — Supplementary Material 1 [file 11136_2026_4189_MOESM1_ESM.docx]

**Supplementary material:**

Title: Understanding Decision-Making Strategies in Discrete Choice Experiment Tasks When Valuing Health States That Include Duration, a Cognitive Interview Study with Australian adults

Contents

[SM1 Pilot interviews 2](#_Toc217414651)

[SM2 Reflexivity 3](#_Toc217414652)

[SM3 Consent & demographic questions 4](#_Toc217414653)

[SM4 Semi-structured interview schedule (sample survey questions and interview prompts) 7](#_Toc217414654)

[SM5 Ethical considerations 23](#_Toc217414655)

[SM6 Demographic characteristics 24](#_Toc217414656)

[SM7 Codes: Themes and sub-themes 25](#_Toc217414657)

[SM8 Table: Themes, sub-themes and example quotes 27](#_Toc217414658)

# SM1 Pilot interviews

Two pilot interviews were conducted on a convenience sample (female aged 77 years, and male aged 49 years) to test the interview prompts and logistics of the interview.

The pilot interviews identified challenges for some participants in moving between the Sawtooth for the DCE questions, Qualtrics for the demographic questions and the Zoom platform.

Following the pilot interviews some simplification was made to the interview process regarding reducing the number of different platforms used during the interview. This resulted in the interviewers sharing their screen and inputting the participants' answers.

Minor changes were made to the interview prompts, which were generally judged to appropriately generate descriptions of the participant’s thought processes.

# SM2 Reflexivity

Both interviewers were female and of a similar age, which may have influenced rapport and interaction with participants. One interviewer was an economist, the other a health outcomes researcher; their shared experience in discrete choice experiment research meant they were accustomed to rational-choice frameworks, so reflexive notes were used to remain aware of any assumptions regarding structured decision-making. Although both interviewers were English, one was based in Australia during data collection, which helped reduce some contextual distance but did not eliminate potential cultural or linguistic differences when engaging with Australian participants. Conducting interviews online also required attentiveness to communication cues and participant comfort. The interviewers met regularly as part of the coding process. This allowed for both discussion and reflection of how their positionality and disciplinary background may influence questioning (i.e., conduct of the interviews) and interpretation (i.e., coding).

# SM3 Consent & demographic questions

Q3 Thank you very much for taking the time to complete this questionnaire. Please complete every question. All information you provide is confidential. The information you give will not be used in any way that could identify you.

Q1 **It is important that you understand what you are being asked to do. You should have received a Plain Language Statement telling you about study. You can look over this again if you click** Information about the study plain language statement **and it will download the file.**
 
**During this interview you will be asked to: firstly, complete background questions about you and your health, and secondly, make some choices between living in different imaginary health states, and living different lives that vary in terms of health state and length of life during this task the researcher will encourage you to talk about what you are thinking and ask questions about how you found doing the tasks.**
 
**It is also important for us to know you are happy to agree to all aspects of the study.**

 
**Please click if you agree with each statement:**

- I consent to participate in this project, the details of which have been explained to me, and I have been provided with a written plain language statement to keep. (1)
- I understand that the purpose of this research is to test the way questions will be asked in a future study to value different aspects of health and score a health questionnaire. (2)
- I understand that my participation in this project is for research purposes only. (3)
- I understand that my interviews will be audio recorded. (6)
- I acknowledge that the possible effects of participating in this research project have been explained to my satisfaction. (11)
- I understand that my participation is voluntary and that I am free to withdraw from this project anytime without explanation or prejudice and to withdraw any unprocessed data that I have provided. (7)
- In this project I will be required to take part in an online interview for one hour with a researcher. During this interview you will be asked to: firstly, complete background questions about you and your health, and secondly, make some choices between living in different imaginary health states, and living different lives that vary in terms of health state and length of life during this task the researcher will encourage you to talk about what you are thinking and ask questions about how you found doing the tasks (10)
- I understand that the data from this research will be stored at the University of Melbourne and will be destroyed 5 years after the final publication. (8)
- I have been informed that the confidentiality of the information I provide will be safeguarded subject to any legal requirements; my personal data (the recording and any personal contact details) will be password protected and accessible only by the named researchers; I understand that my anonymised data may be shared with other researchers. (9)

Q4 How old are you?

________________________________________________________________

Q5 What is your gender?

- Male (1)
- Female (2)
- Gender diverse / other (3)
- Prefer not to say (4)

Q17 What is your highest qualification?

- Bachelor degree or above (1)
- Advanced diploma or diploma or certification (III, IV) (2)
- No non-school qualification (3)

Q6 How would you describe your ethnic group?

________________________________________________________________

Q7 Have you experienced serious illness

|  | Yes (1) | No (2) |
| --- | --- | --- |
| in yourself? (1) |  |  |
| in members of your family? (2) |  |  |

***** Respondents then completed the EQ-5D-5L (English version) electronically (i.e., self-complete). The EQ-5D-5L is not shown due to copyright reasons. *****

# SM4 Semi-structured interview schedule (sample survey questions and interview prompts)

**Title:** A qualitative study to explore methods to value and score a health questionnaire

[Note interviewer explanation and probes are show in **blue**]

*Interviewer:*

- *Welcome and thank you for taking part*
- *Introduction to researcher*
- *Before we begin the interview, I would like to make sure that you understand what you are going to be asked to do, and that you are happy to agree to take part in all aspects of the study*
- *I would first like to ask whether you are happy to have this session recorded – it isn’t being recorded yet but I would like to start the recording once you have agreed to this.*
- *Thank you – I’m now going to turn on the recording. If you have your camera turned on this will record both sound and what is showing on the screen, however, immediately after the video we will delete the visual recording and keep only the audio recording.*
- *In the email we sent a summary of what will happen in the research - we call it a Plain Language Summary. Would you like to read this again, or I can talk you through each section if you like?*
- *Do you have any questions you would like to ask about the interview or the research?*
- *It is important that you know that all the information you give in this survey is anonymous – we won't link it to your name in any way*
- *I’m now going to ask you to read each statement and tick it if you are happy to agree with the statement.*
- *Thank you.*

*For the first part of the interview, I’d like to ask some background questions about you and your health*

*We ask these questions for two reasons*

1. *so we know who we have spoken to and how representative our sample is to the Australian population*
2. *because we would like to understand whether decisions people make about imaginary health states differ according to their own health and characteristics (e.g. do younger people interpret our questions differently to older people?)*

Introduction displayed on the screen:


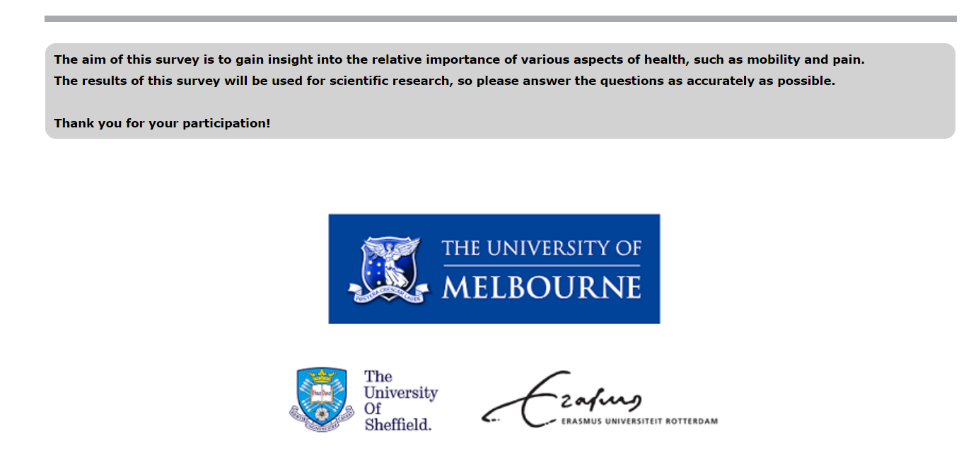


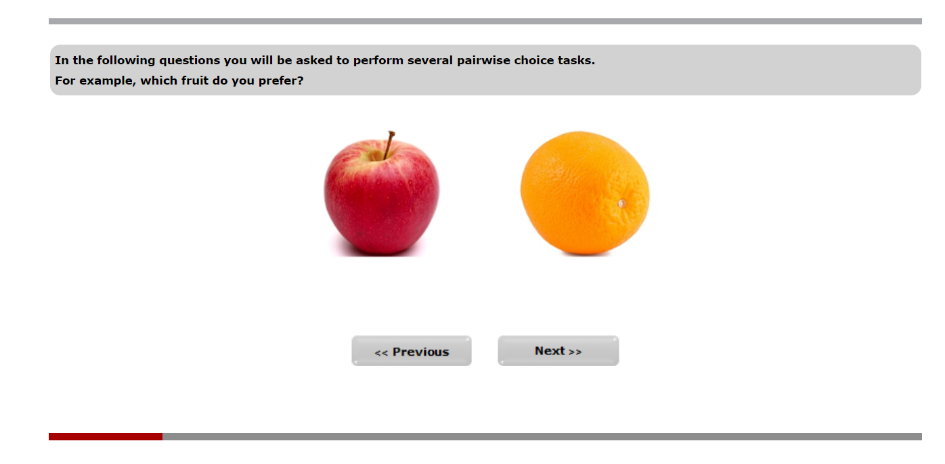


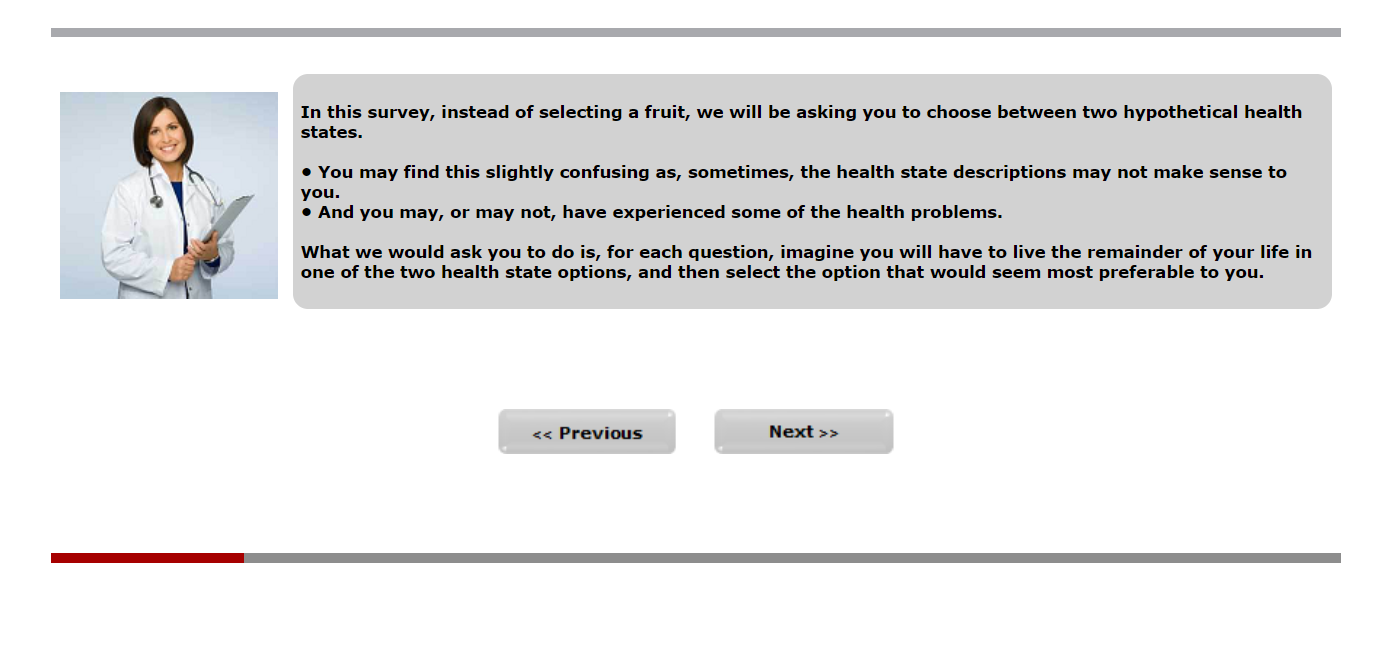


***Interviewer*** *– these are practice questions so you can get a feel for the survey and how questions look*

PRACTICE 1


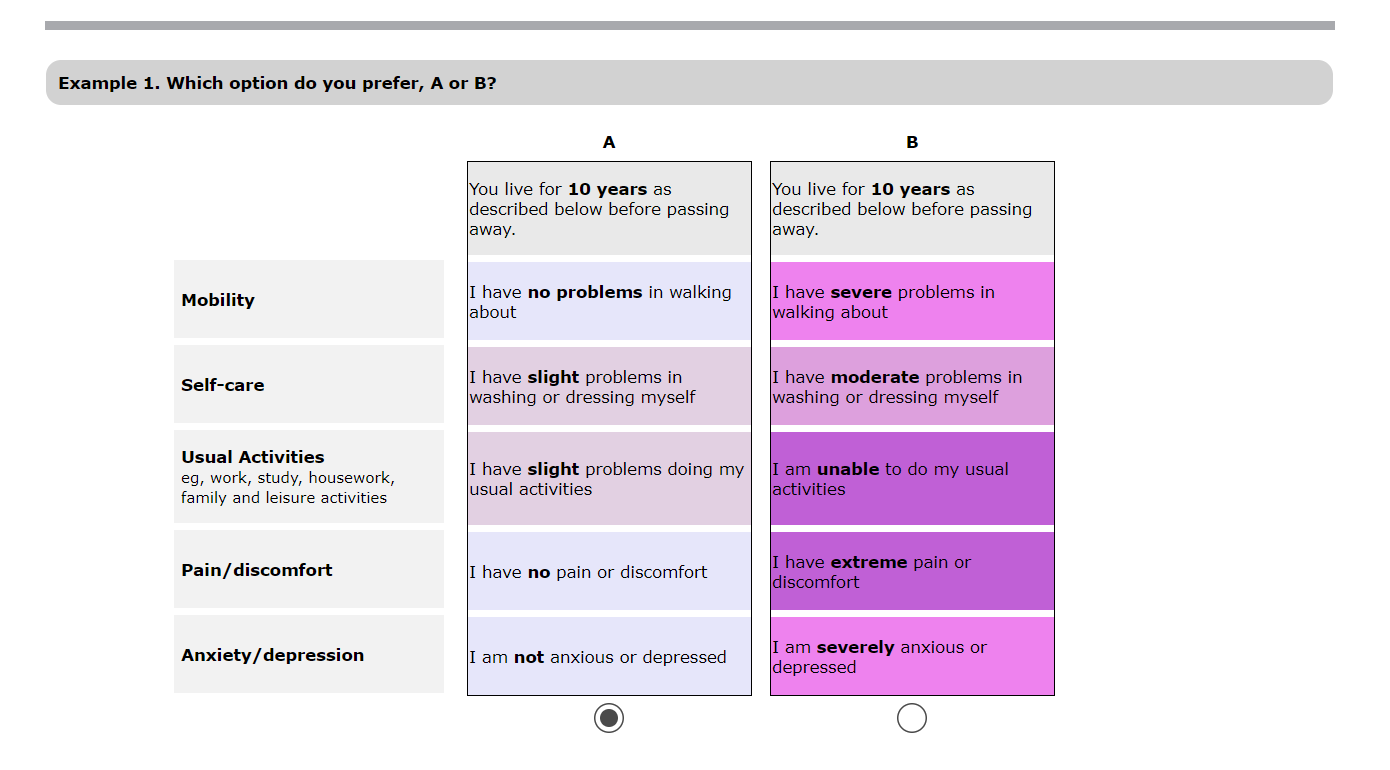


***Interviewer*** *– Was it clear what you had to do?*

*OK let’s do another practice one*

PRACTICE 2
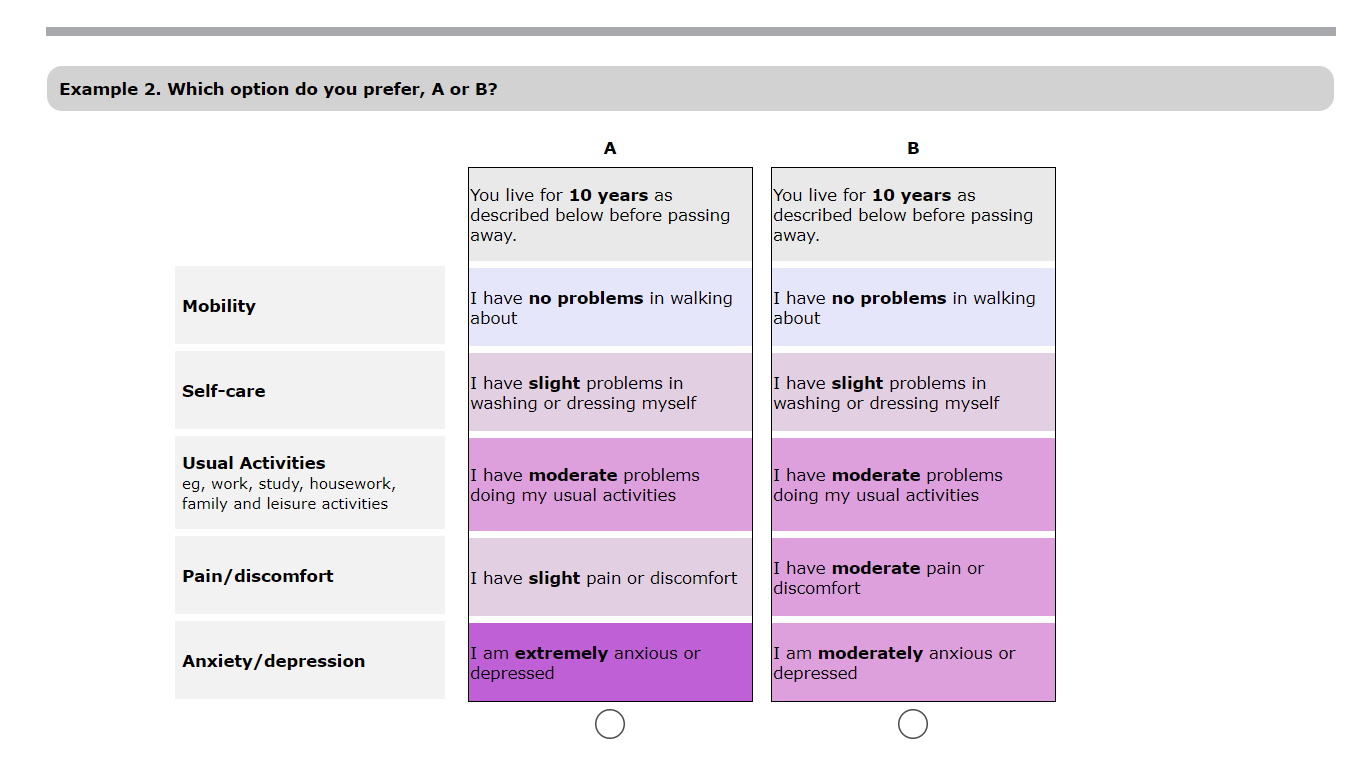


***Interviewer*** *– Was it clear what you had to do in this one?*


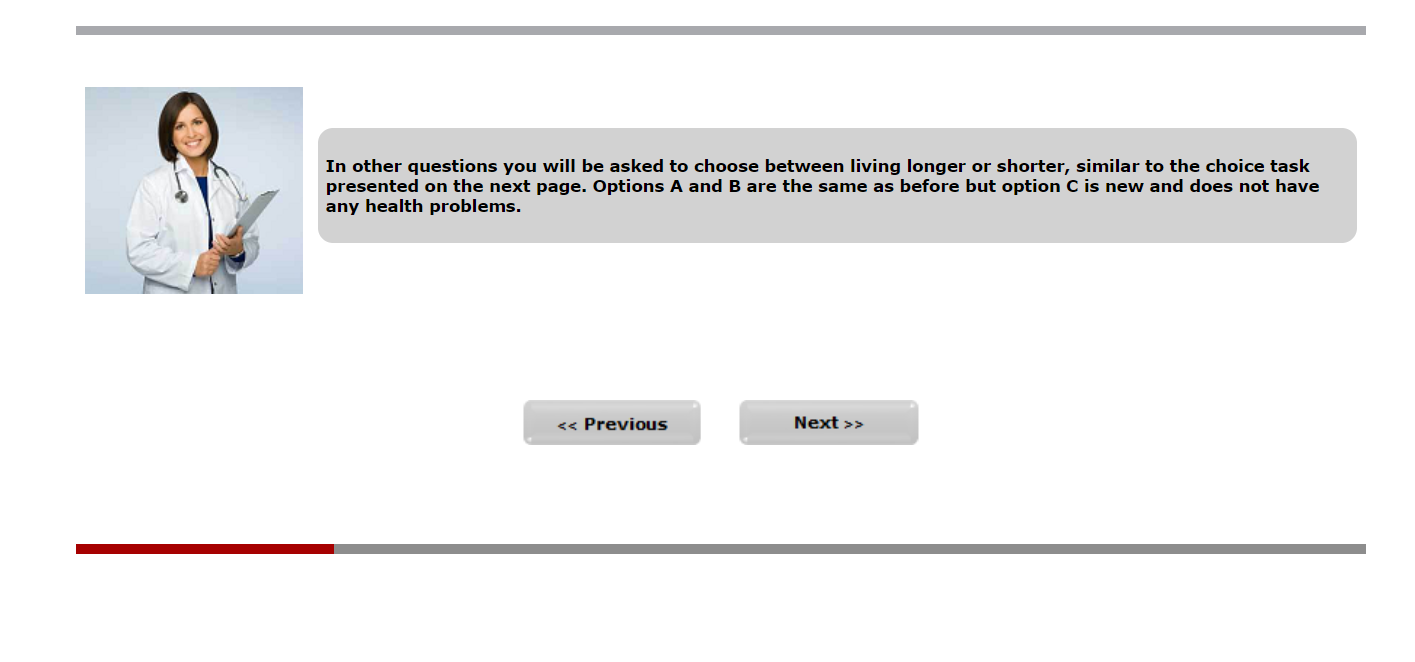


QUESTION 1


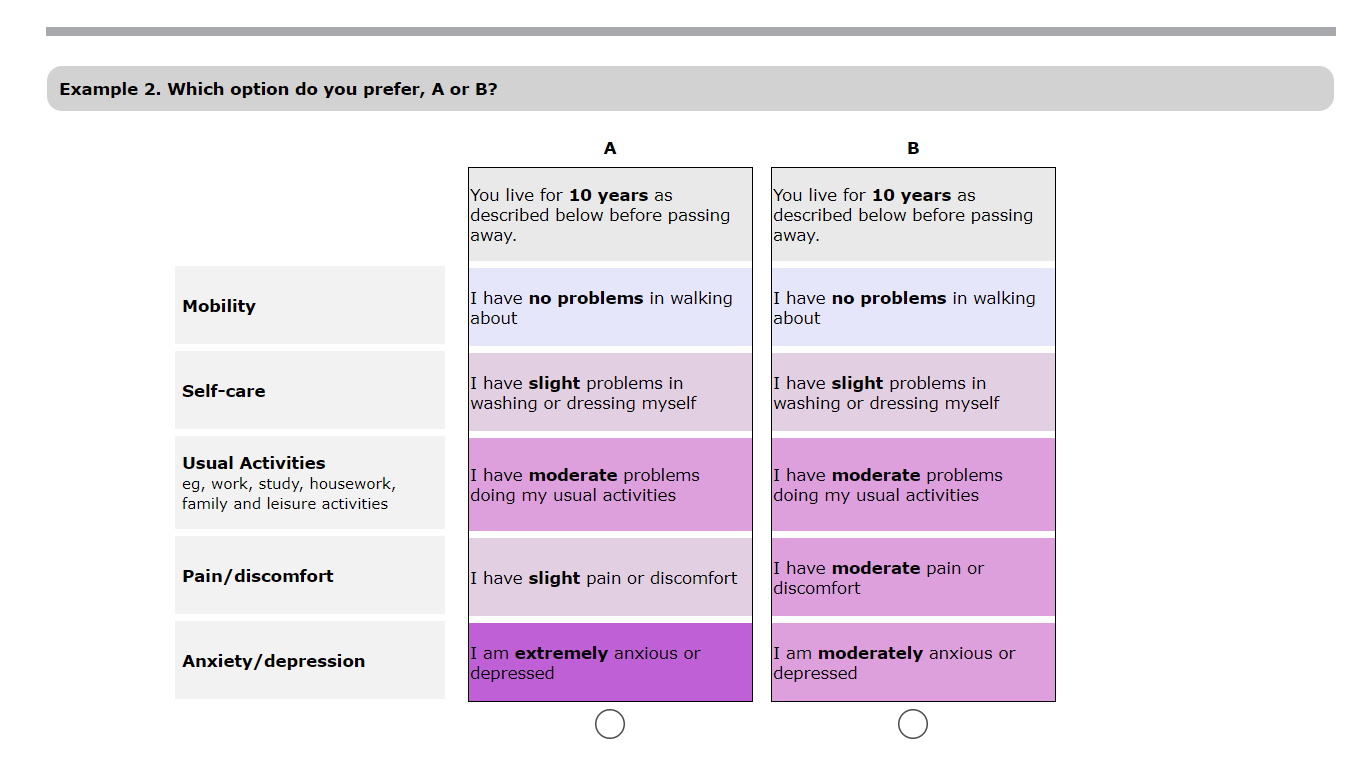


Note: additional questions were presented in which the durations within the choices above varied e.g. 10 years vs 8 years.

***Interviewer:***

- *Can you tell me something about how you made your decision?*
- *What did you think about the colours? Were they helpful or confusing?*
- *Did you think about each aspect in turn and imagine living for 10 years with that aspect (so having slight problems in washing or dressing for 10 years)– or did you just compare each in turn (the level of problems in washing dressing for A vs that for B)*
- *How difficult was this decision?*


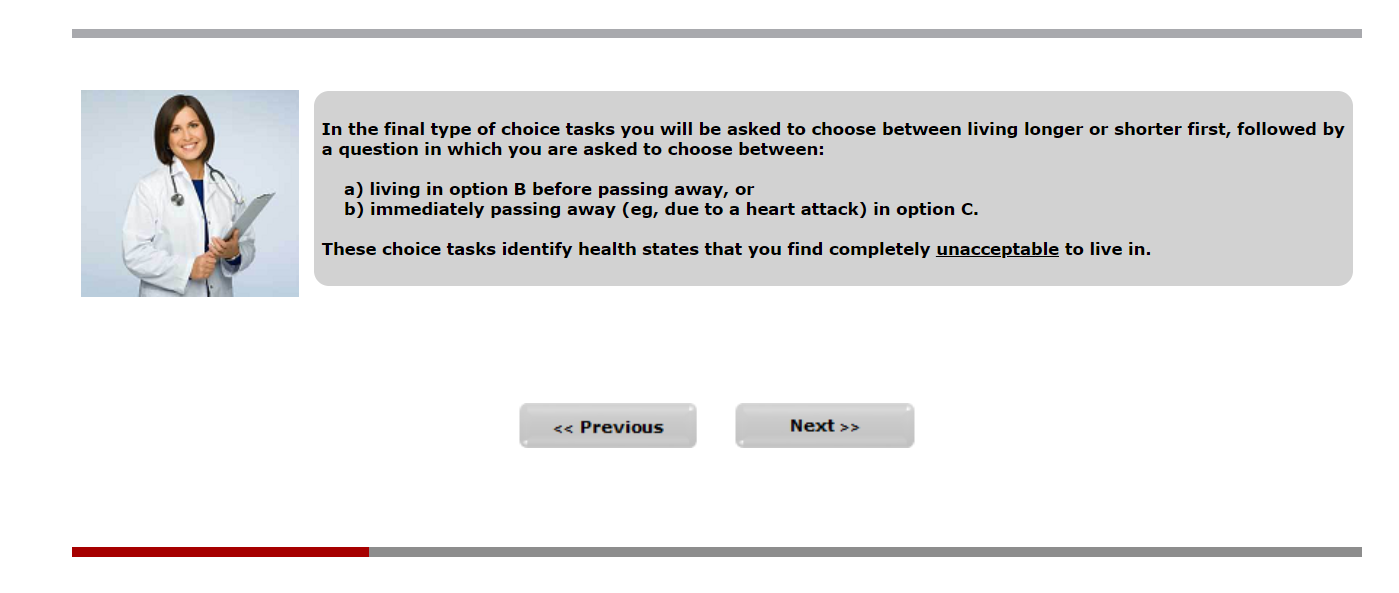


QUESTION 2


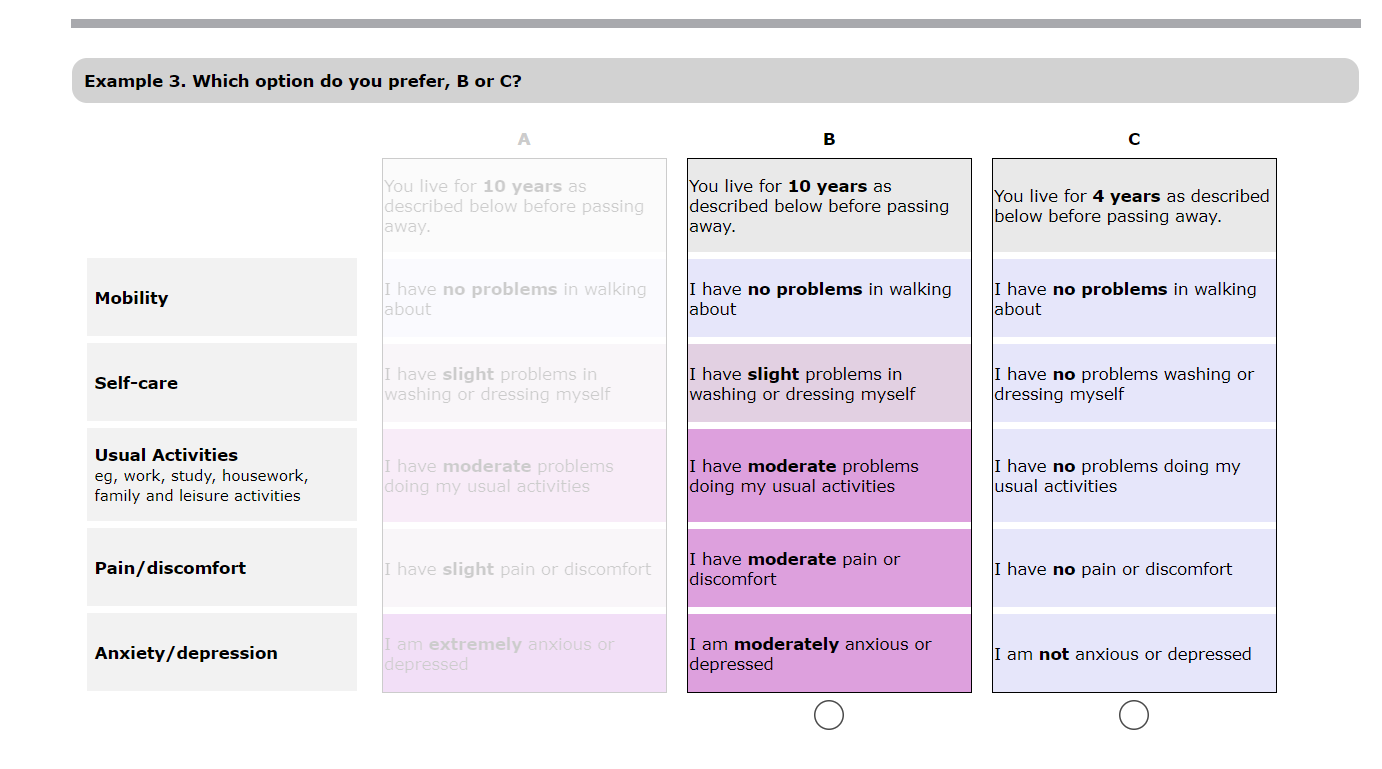


***Interviewer***

- *Can you tell me something about how you made your decision?*
- *How difficult was this decision?*
- *How did you feel about giving up years of life?*

QUESTION 3


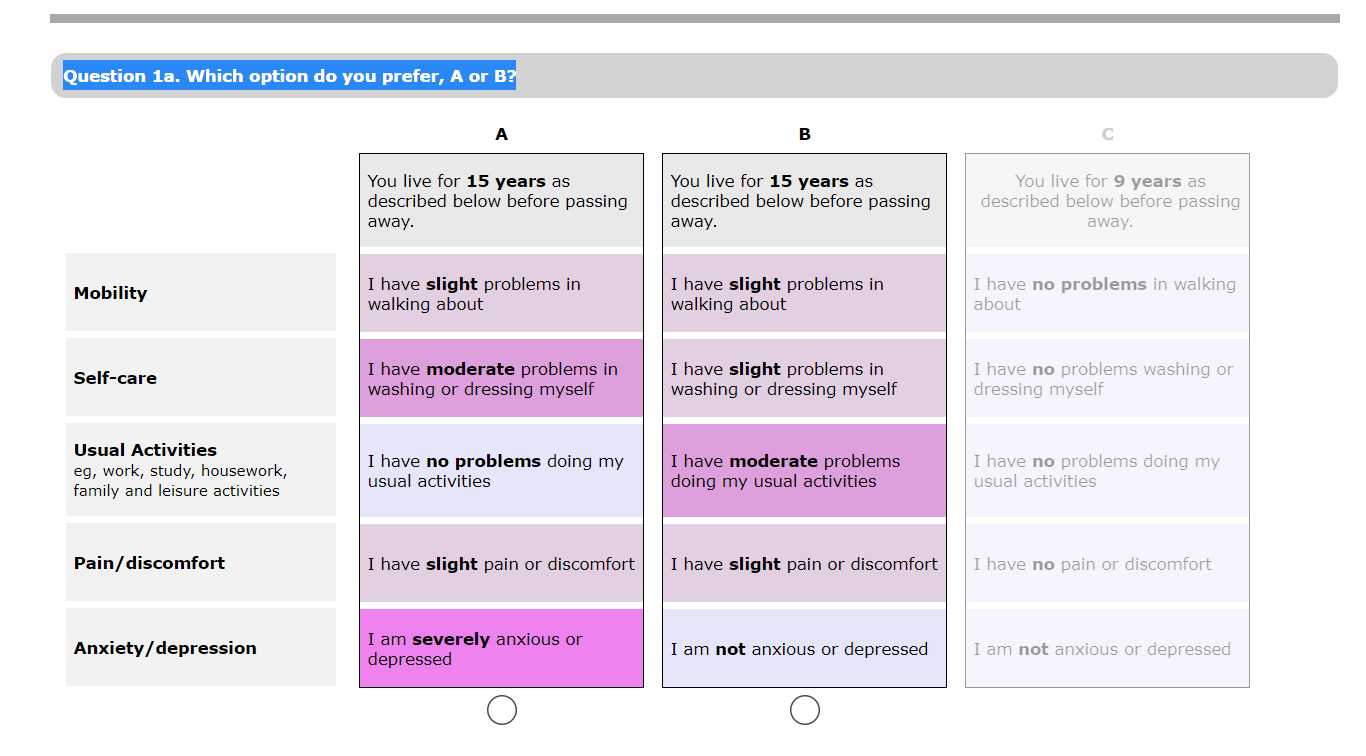


***Interviewer***

*Can you tell me a bit about how you made that decision?*

*Did you notice that some of the domains were the same between the 2 options? Did you still use those in your decision making?*

*Do you think it makes it easier to make a choice when one of the aspects is the same between two lives?*

*Did that seem a difficult or easy decision to make?*

*Do you think saying ‘before passing away’ is better than saying ‘dying’ – or does it not matter?*

QUESTION 4


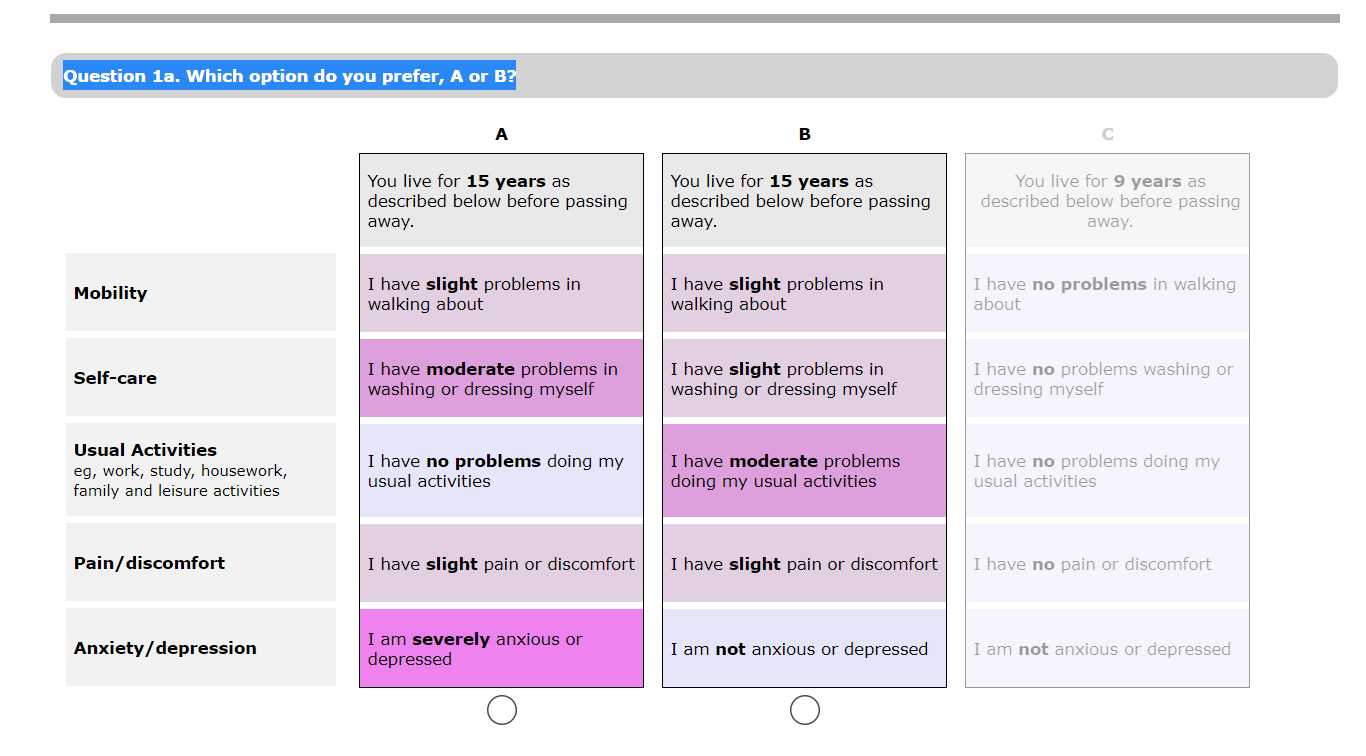


Note: additional questions were presented including differences in duration of 18 vs 15 years.

***Interviewer***

*Can you tell me a bit about how you made that decision?*

*Did that seem a difficult or easy decision to make?*

*Did you notice the duration difference?*

*What did you think about the duration difference (18 vs 15 yrs)*

*Some people make the decision by comparing one aspect at a time between the 2 different lives (so – e.g. no problems with mobility versus moderate problems and then comparing the differences in years lived (so - 15 years vs 18 years). Others look at all the information about the health state and imagine living in that state for 15 years given and compare that to the other health state and imagine living in that state for 18 years. Which method do you think best describes how you made the decision?*

*Did you notice that pain and discomfort was the same for the two lives? Did you take that into consideration or did you just ignore that aspect?*

*[probe into whether they considered the level of the fixed item with the duration given]*

QUESTION 5


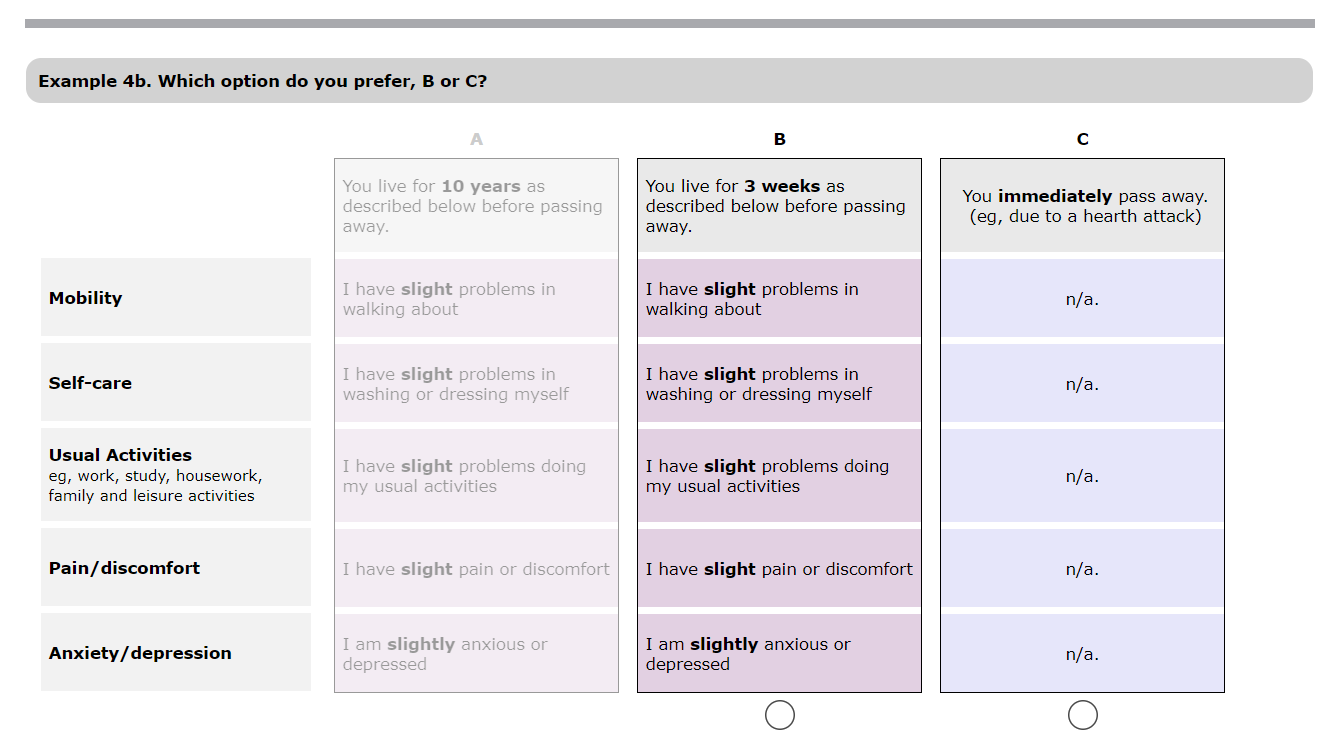


***Interviewer***

- *Can you tell me a bit about how you made that decision?*
- *Does 3 weeks seem quite different to dying immediately?*
- *How did seeing the words ‘immediately pass away (e.g. due to a heart attack) make you feel?*
- *Would it make a difference if it said ‘you immediately pass away’ without adding the explanation or ‘pass away peacefully in your sleep’ or ‘not live any further time at all’*

QUESTION 6


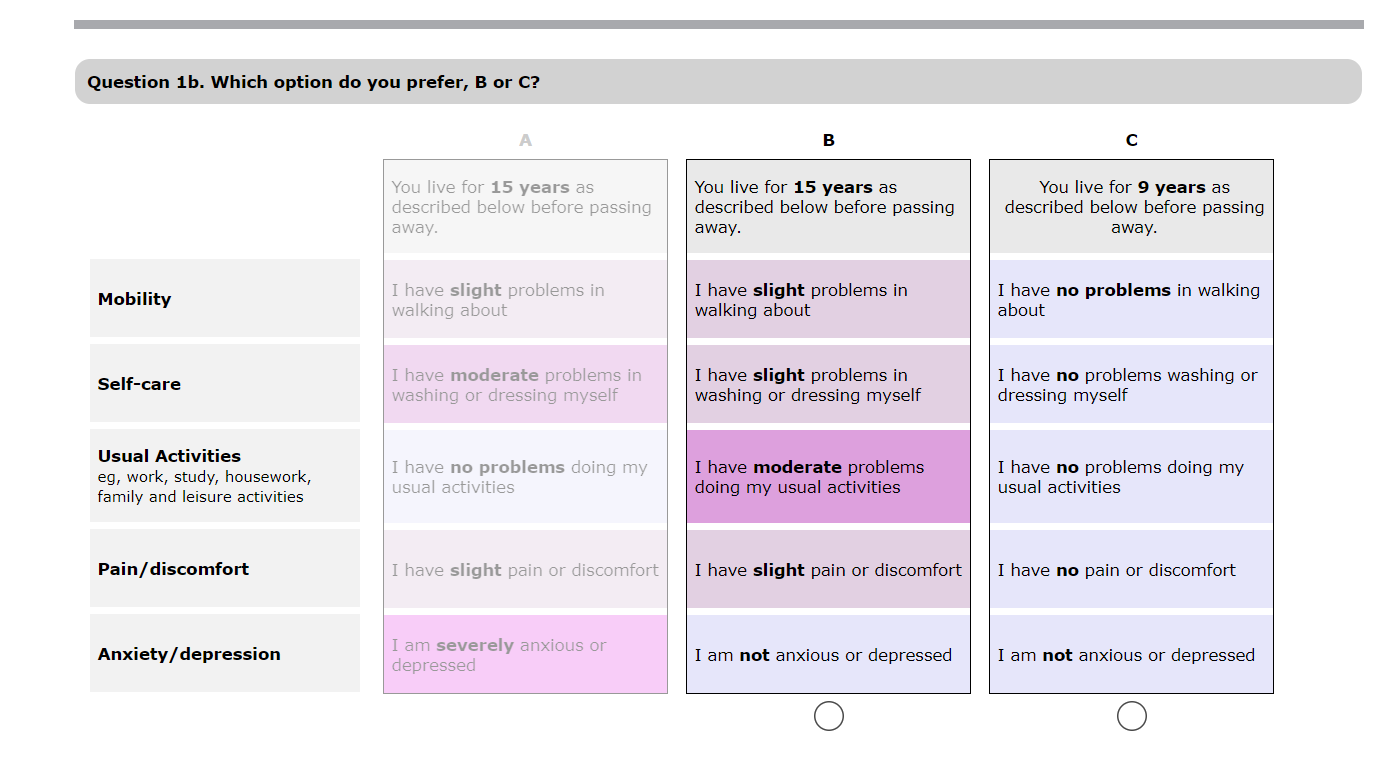


***Interviewer****: Can you tell me a bit about how you made that decision?*

*Did that seem a difficult or easy decision to make?*

*How did you feel about the idea of giving up years of life?*

*[Interviewer to probe around the impact of the duration given]*

*Is there any number of ‘duration’ years that might make you choose the alternative option? [note respond to their choice here]*

*If it said 30 for A and 18 for C which would you have chosen?*

*Probe – is it easier to give up the time when it comes a long time into the future*

QUESTION 7

*
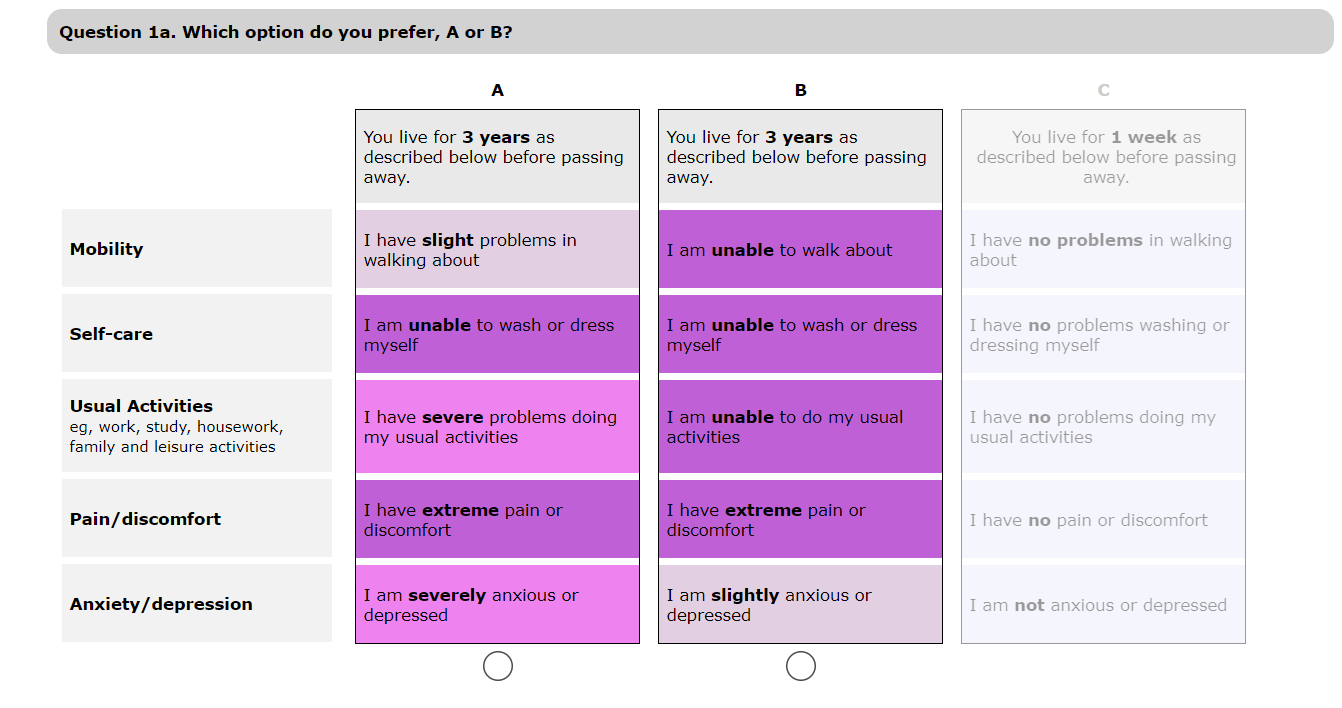
*

***Interviewer:***

*Can you tell me a bit about how you made that decision?*

*Did that seem a difficult or easy decision to make?*

*When you have a poor state like B – would it be better to live for more time – say 5 years rather than 3?*

If the participant opted for shorter time –

*If you could find out something else about these lives (A & B) to help you make a decision about whether you would want to live in it for a long period of time – what would it be?*

*QUESTION 8*

*
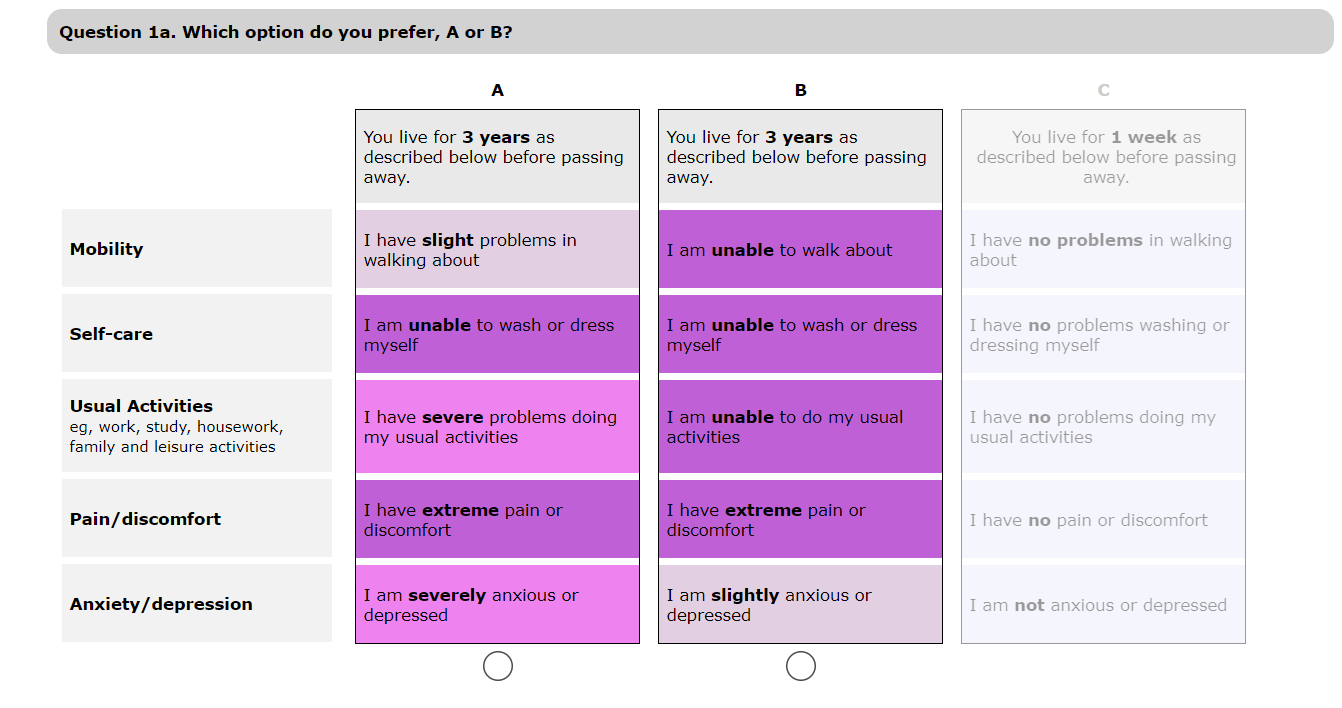
*

Note: additional questions were presented including differences in duration of 3 vs 2 years.

***Interviewer***

*Can you tell me a bit about how you made that decision?*

*Prompt – explore question of whether there is an amount of time they think they could put up with the health state*

*Is it ever the case that additional time wouldn’t make the state more attractive?*

*QUESTION 9
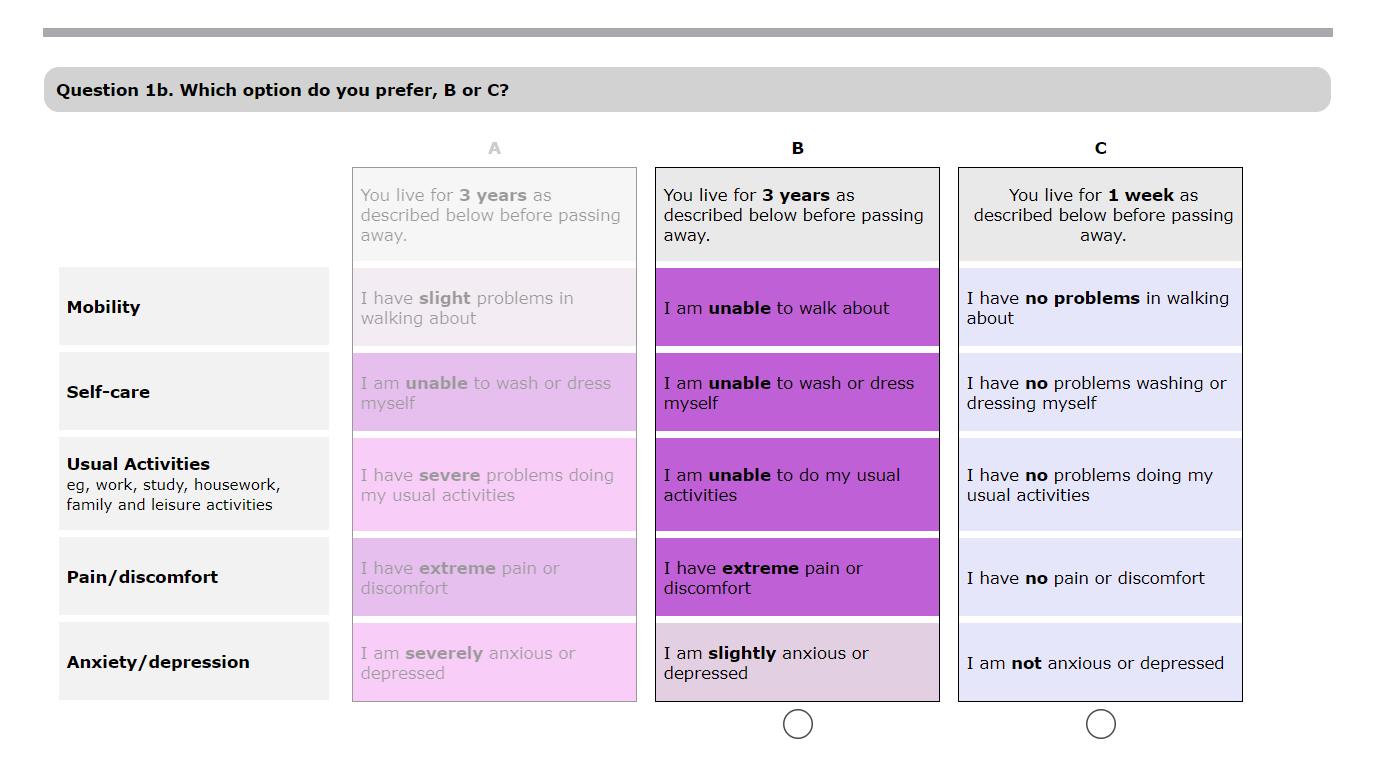
*

***Interviewer:***

*How do you feel about living a short life compared to living longer but with virtually no quality of life?*

*Does 1 week feel much different to passing away immediately*

*QUESTION 10*

*
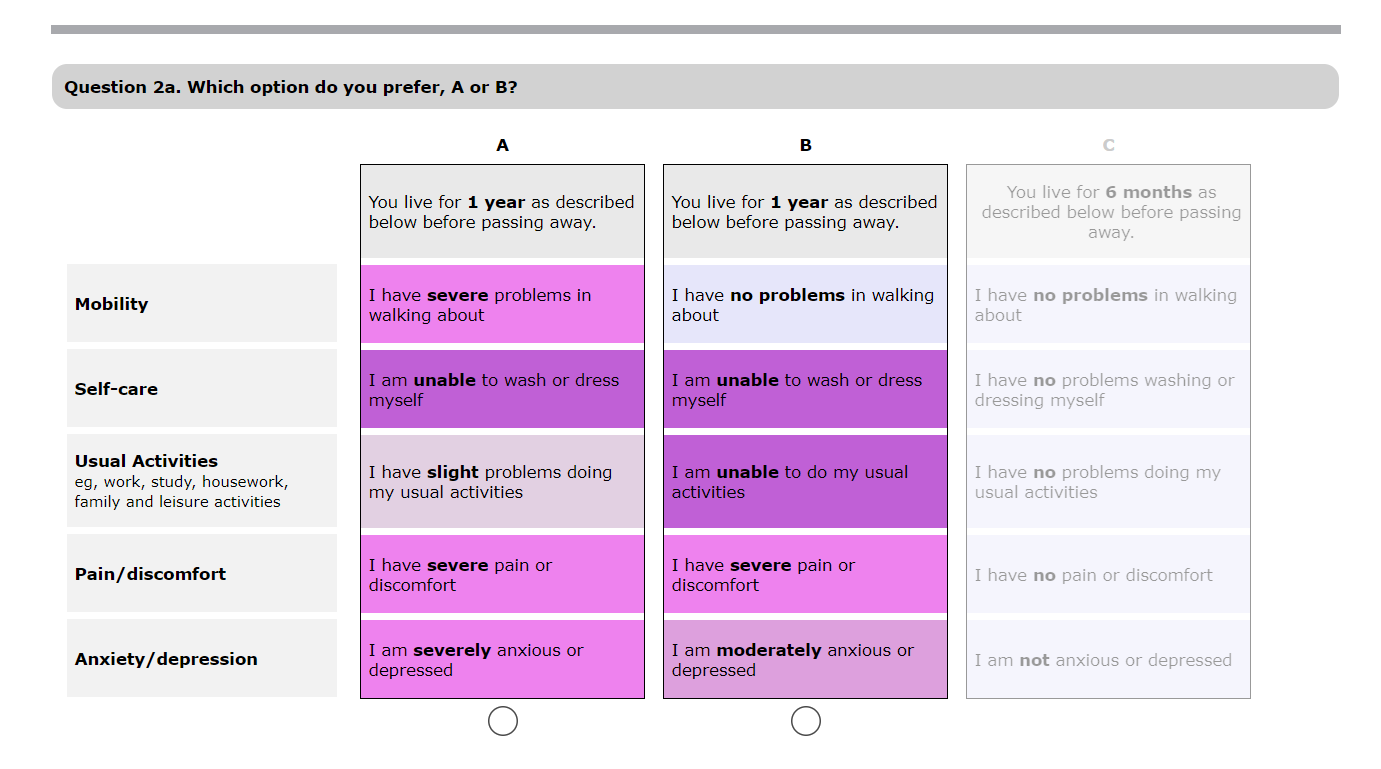
*

***Interviewer:***

*How do you feel about comparing these different states – when they are both very poor states?*

*QUESTION 11
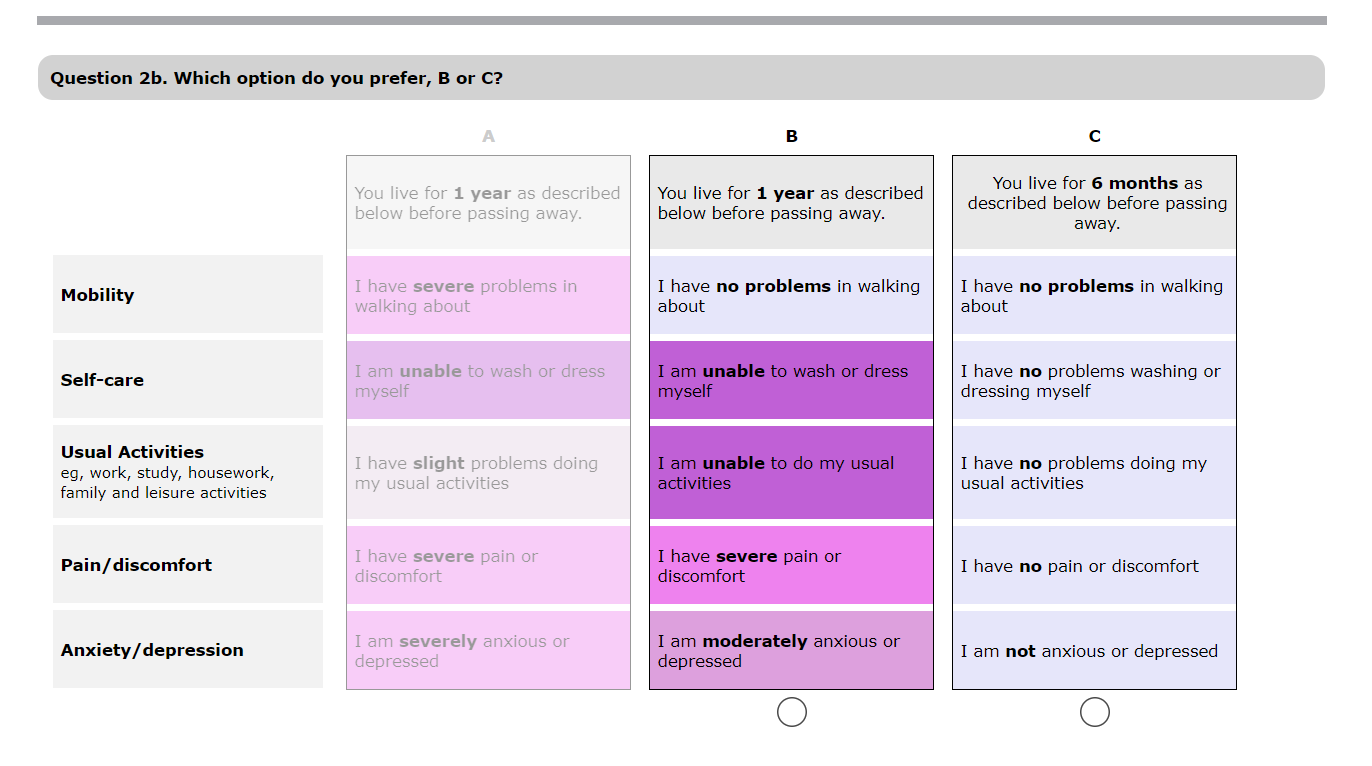
*

***Interviewer***

*Can you tell me a bit about how you made that decision?*

*QUESTION 12/13*

Note PowerPoint also displayed 1 year in the poor health state vs 6 months in the same state and 6 months in the poor state vs immediately pass away

*Interviewer: Can you tell me a bit about how you made that decision?*

*QUESTION 14*

*
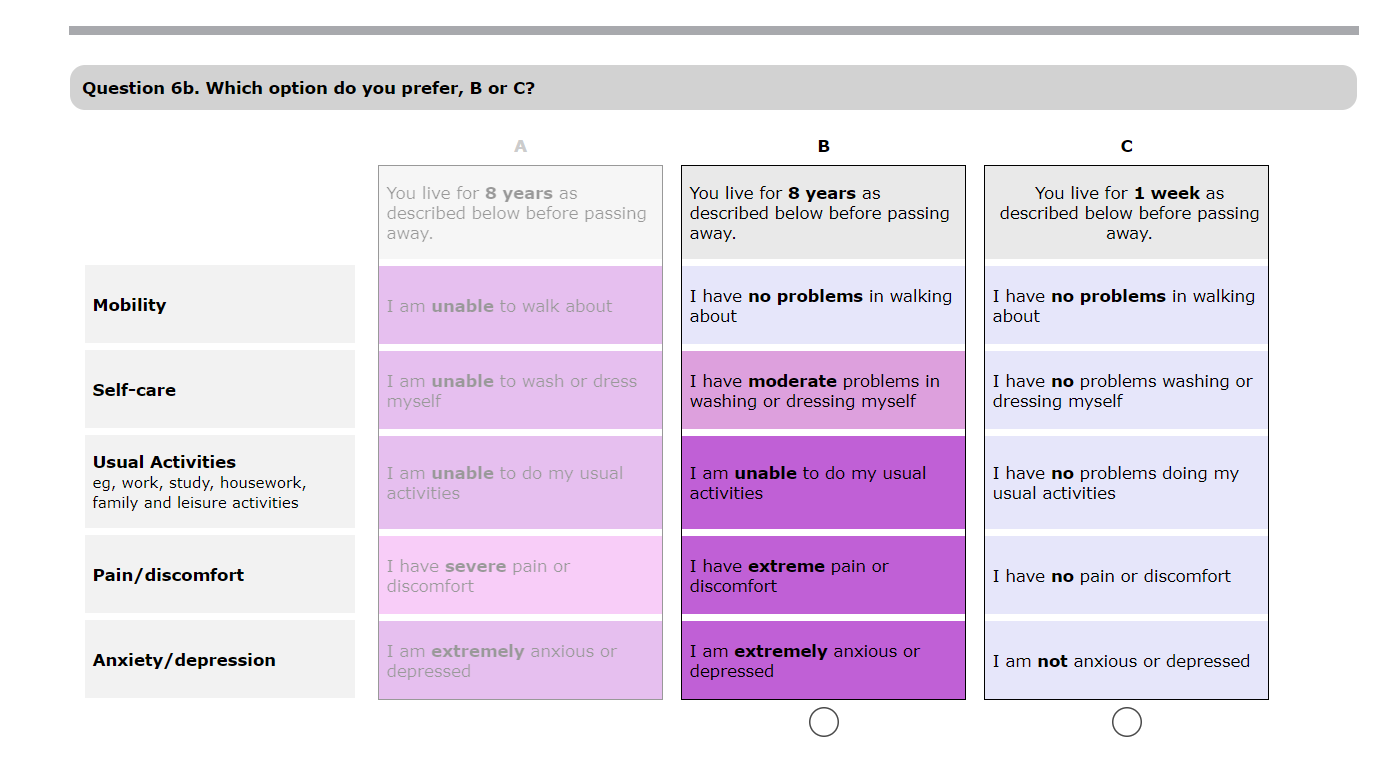
*

***Interviewer:***

*Can you tell me a bit about how you made that decision?*

*Does 1 week feel different to passing away immediately?*

QUESTION 15

*
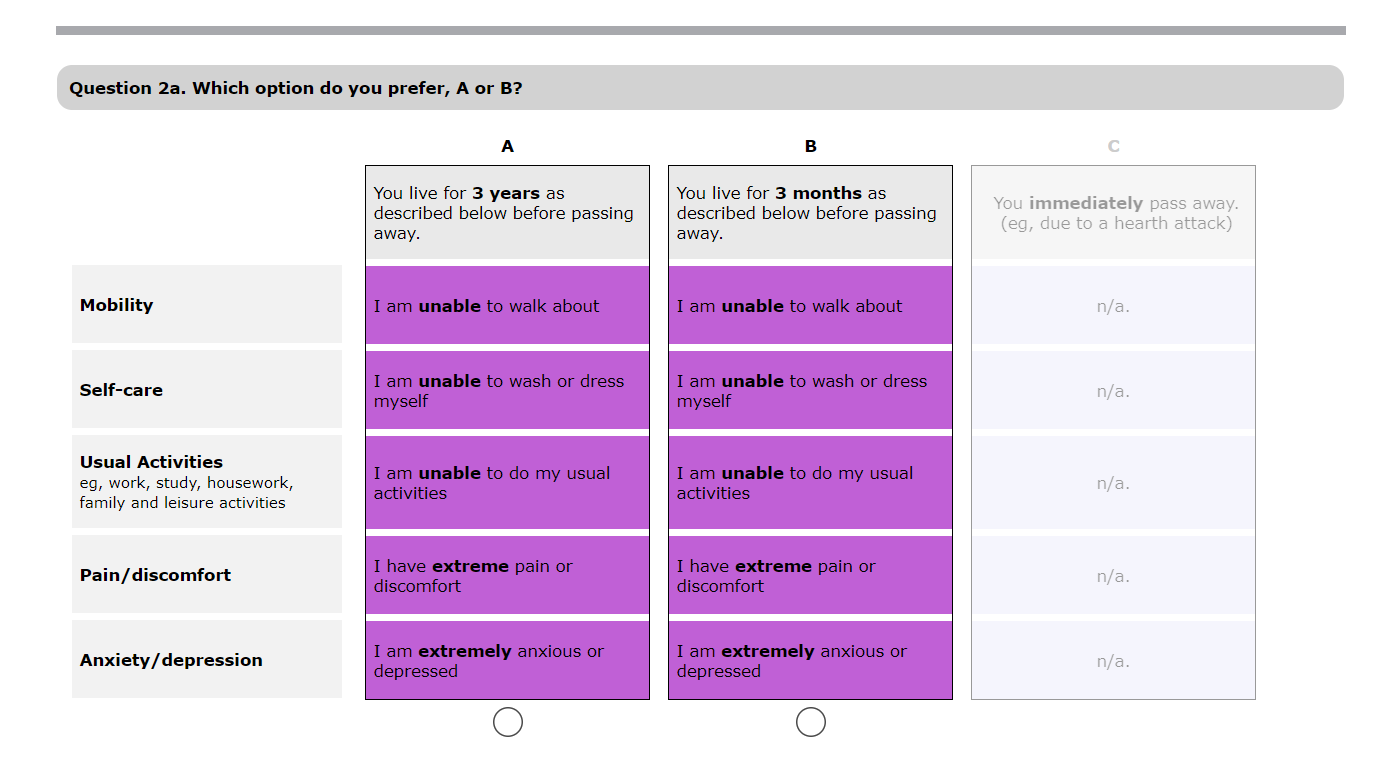
*

***Interviewer:***

*Can you tell me a bit about how you made that decision?*

*Does this seem an easy or difficult decision to make?*

*Can you imagine a health state which you think would be worse than being dead?*

*Can you imagine a health state which you think would be better than being dead if you lived it for just a few years but you wouldn’t want to live in for too long?*

*QUESTION 16*

*
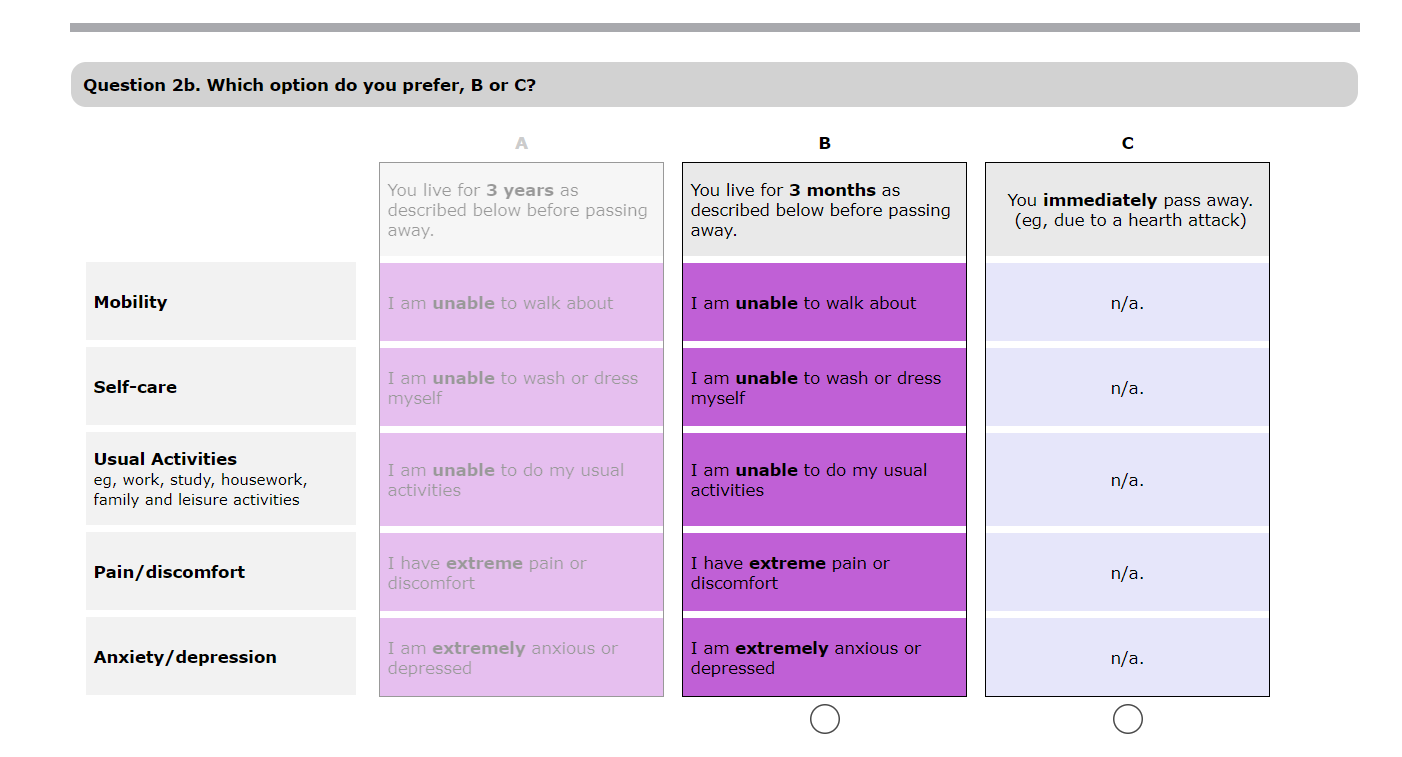
*

***Interviewer:***

- *Can you tell me a bit about how you made that decision?*
- *When you see the phrase ‘immediate death’ what springs to mind?*
- *How does the example of ‘heart attack’ make you feel?*
- *Would it make any difference if another example/no example was given?*
- *Does this seem an easy or difficult decision to make*?

**Interviewer**

- draw to a close
- thank you
- process for reimbursement

# SM5 Ethical considerations

Presenting participants with choices involving immediate death can be distressing. The interviewers were experienced researchers who have both dealt successfully with participants experiencing distress during interviews. This includes heightened observation to identify when participants may be distressed, patience to allow participants to take their time, recognising when to offer to stop the interview (ensuring they are still rewarded even when interviews are terminated early), and gently managing the close of the interview. The ‘Plain Language Statement’ describing the interview also provided resources that participants could access, and researchers highlighted these to participants who displayed any indication of distress.

Transcripts were anonymised and only linked to demographic survey through date and time of the interview. In line with participant consent, data was not shared outside of the study team. All data will be deleted five years after publication.

Ethical approval for the study was granted by the University of Melbourne’s Human Research Ethics Committee (ID 2057011.1).

# SM6 Demographic characteristics

| **Participant characteristics** | **Frequency/Mean** |
| --- | --- |
| Gender: Female | 9 (42.9%) |
| Male | 12 (57.1%) |
| Age: Mean (SD), range | 53.3 (14.7), 29-75 |
| EQ-5D-5L score*: Mean (SD), range | 0.877 (0.141), 0.517-1 |
| Experienced serious illness personally | 5 (23.8%) |
| Experienced serious illness within the family | 11 (52.4%) |
| Highest qualification: |  |
| Bachelor’s degree or above | 8 (38.1%) |
| Advanced diploma or diploma or certification (III, IV) | 8 (38.1%) |
| No non-school qualification | 5 (23.8%) |
| Ethnicity: |  |
| Australia Caucasian | 16 (76.2%) |
| Asian | 3 (14.3%) |
| Eastern European | 2 (9.5%) |

*** EQ-5D-5L score based on Norman R, Cronin P, Viney R. A pilot discrete choice experiment to explore preferences for EQ-5D-5L health states. Appl Health Econ Health Policy. 2013 Jun;11(3):287-98, calculated using STATA 16*

# SM7 Codes: Themes and sub-themes

**Theme: Factors influencing decision-making**
*Key codes:*

- Willingness to trade life years
  - Reluctance to trade due to desire to be with loved ones
  - Reluctance to trade due to potential for a future cure
  - Reluctance to trade due to view of life as inherently valuable
  - Willingness to trade due to desire to prioritise good quality of life
  - Willingness to trade due to concern over being a burden to others
  - Willingness to give up all time for states judged worse than dead
- Maximal endurable time
- Value of time in the final weeks of life
  - Judges very short durations as similar
  - Very short durations of time (1 to 3 weeks) judged differently to immediate death
- Valuing time in the near or far future
  - Valuing time in the future different due to expected health state
  - Anticipating different preferences for the elderly
  - Differences between 8 and 10 years, 15 and 18 years not important
  - Treating time differences as absolute value

**Theme: Process of decision-making**
*Key codes:*

- Interpreting the health state and choice offered
  - Drawing on own experience (self or others) of the state
  - Hard to value health attributes in which experience is lacking
  - Some states judged to be unrealistic
  - Hard to value unrealistic states
  - Lack of confidence in their decision as missing details on medication/treatment
  - Lack of confidence due to uncertainty in ‘usual activities’
  - Lack of confidence in their decision as it is only hypothetical
  - Impact of a health attribute varies depending upon the level of other health attributes
- Interactions between duration and health state: Multiplicative or additive thought processes
  - Multiplicative approach: Considering health attributes in combination with the duration
  - Additive approach: considering health attributes and the duration attribute separately without consideration of their interaction
  - Additive approach: ignoring health attributes that are similar when durations differ

**Theme: Survey design**
*Key codes:*

- Wording around the ‘dead state’
  - ‘Passing away’ gentler, softer, kinder, more polite than dying
  - ‘Passing away’ infers a different type of death to dying
  - Happy with any language
  - Preferred the term ‘dying’
  - The term ‘dying’ easier for participants with English as a Second Language (ESL)
  - Emotive response from participants due to confronting language
  - Participants disliked the phrase ‘not living any time at all’
  - Recommendations for other terms
  - Interpretation of ‘dying immediately’
- Use of colour-shading and bolding of text
  - Participants liked the use of colour
  - Colours make decision making easier
  - Heavy reliance on colour
  - Indifference to colours
  - Meaning of colours unclear
- Potential errors and inconsistencies
  - Overlooked the difference in years in choice task
  - Selected choice closest to own health
  - Tried to select the greyed-out choice
  - Confused by the option ‘being dead’
  - Health state is fluid during discussion
  - Health state is not considered constant
  - Inconsistent choices arising due to change in focus

# SM8 Table: Themes, sub-themes and example quotes^^[[1]](#footnote-1)^^

**Theme: Factors influencing decision-making when there is a trade-off between length of life and quality of life**

| **Sub-theme** | **Description** | **Example quotes (#)** |
| --- | --- | --- |
| **Willingness to trade life years for improved health** | Reluctance to trade due to desire to be with loved ones | 1. *“Giving up 6 years – that’s a lot. That’s 6 years with your kids, with your family"(ID1)* 2. *“I’d want to live as long as possible even If I’ve got to put up with elements." … "it might be me not being to do all the housework as effectively as I used to but I’m still here for 15 years to see grandkids grow up and such” (ID5)* 3. *“because I have a new grandchild and a baby and a whole family of people wanting me to hang around- so I probably would have to choose option A” (ID9)* 4. *because if you have loved ones 2 more years is a lot actually” (ID11)* |
|  | Reluctance to trade due to potential for a future cure | 1. *“I would prefer to live 10 years honestly – because science is going ahead – medical science is going ahead – I would live with the hope that tomorrow will be a better day and positive changes will happen” (ID7)* 2. *“I work on the assumption that where there is life there is hope” (ID9)* 3. *“I think the unspoken – unpresented part that comes into that is that the longer the time period the more chance or more possibility is that these could be alleviated through cures or through development” (ID21)* |
|  | Reluctance to trade due to view of life as inherently valuable | 1. *“each day is valuable – as many as possible” (ID7)* 2. *“I value human life – I value animal life – I daren’t tread on a daffodil or something like that without thinking of my god maybe I shouldn’t have done that – and human life is important to me – that’s why I’ve spent all the years of my life maintaining that – I wouldn’t’ like to put a cost benefit on that” (ID9)* |
|  | Willingness to trade due to desire to prioritise good quality of life | 1. *”...it's a shorter life span but at least I’m living a more happier healthier lifestyle.” (ID2)* 2. *“I value having no problems for a shorter period than having 10 years of extra live and being miserable" (ID10)* |
|  | Willingness to trade due to concern over being a burden to others | 1. *“not being a problem for anybody” (ID2)* 2. *“You’d feel – you wouldn’t’ want to say it but you’d feel a burden to the person who has to care for you and do all the stuff – it's not what they signed up for” (ID5)* 3. *“I would take C – the reason is the independence and its very – painful mentally to be depend on others – and have carers who would be coming and helping in basic activities” (ID7)* |
|  | Willingness to give up all time for states judged worse than dead | 1. *“I am unable to wash and dress myself, severe.. I mean I think I’d much rather just go” (ID16)* 2. *“I’d rather just be euthanized” … “The quality of life isn’t there – and the moment the quality of life is compromised – 4 out of 5 – I don’t think there is much to live for really." (ID2)* 3. *“We’ve just brought in euthanasia to this state so I think I’d opt for that. I don’t’ think there is any quality of life there at all – you can’t wash or dress yourself – extreme pain – slightly anxious other one severely” (ID18)* 4. *“I certainly wouldn't’ like to be living in those states – I think if I was living in A I would prefer to be dead” …”you have to live 3 years basically unable to do anything” (ID17)* |
|  | | |
| **Maximum Endurable Time (MET)** | Describes MET preferences | 1. “It’s all those ‘unables’ - all that care that you can’t do for yourself you’ve got to entrust someone and you wouldn’t w*ant to do that for years and years and years" … “I’ll hack it out for 6 months but I can’t do that for 8 years" (ID5)* 2. *“In that scenario [note: 6 months in 15543 vs immediate death] – It's only God damn 6 months – I think I would try and probably soldier on for the 6 months knowing that I was going to pass away at the end of it" … “If you said a year I’d be going Oh my God but 6 months – 3 months would have been even better – 6 I can cope with I think – 6 months would fly by” … “you think you can deal with anything but eventually – constant pain over a period of time will get to you” (ID10)* 3. *"maybe by the time you live 15 you might think OK – I’ve had enough of this" with the wrong mental state I don’t know whether 5 years is bearable – I don’t even know whether 3 years is bearable" …”If you are told you only have 6 months and then you will be thinking OK I still have my loved ones I can still spend that time with them so you are thinking OK I can bear with this – just for that short period of time – to have that time with them. I don’t know whether some people think it is more for themselves or more for the people you leave behind” (ID11)* 4. *“[choice set of state 555552 for 3 years or 2 years] Honestly maybe the 2 years is a little bit better because it's 2 years and you would not suffer as much… [choice set of 55552 for 3 years or 1 week] If it was 6 months not 1 week I would take that – but 1 week is really very short” (ID7)* 5. *I haven’t experienced it but I do know people with chronic conditions – about 18 months to 2 years that would be the tipping point – from what I’ve heard from others” (ID21)* 6. *“Both got terrible pain. I wouldn’t want to live longer than 3 years in that case.” (ID1)* 7. *"I don’t think personally I would like to live my live for the next 8 or 10 years in extreme anxiety – I think it would just wear you down mentally, physically" (ID9)* 8. *“I think that the problem that I would have in A, because of my past experience, I wouldn’t want to live years and years and years”. (ID16)*   *Others, when asked, gave examples of possible scenarios in which a state would be valuable to live in but only for a short period of time,*   1. *"yes, look if there is something I want to see happen – if I had children or something and I wanted to see them graduate or you know do something then sure" (ID3)* |
|  | | |
| **Value of time in the final weeks of life** | Judges very short durations as similar | 1. *[Immediate v 3 weeks] “I would say similar” (ID20); [1 week vs immediately] but I don’t think there is a big enough difference for me to really consider it (ID13)* 2. *"3 weeks is really nothing" (ID2)* |
|  | Very short durations of time (1 to 3 weeks) judged differently to immediate death | 1. *[Immediate death vs 1 week] “I have to look at it differently” (ID8)* 2. *"more to say goodbye to family and friends – I’ve got my house in order – I’ve got a will and I’ve got that sorted - I might want to use the time in terms of documenting – how to get into my computer and if they need to access finances – and give them passwords – and all sorts of crap like that- they are going to have to execute the will so I’d rather they had all that information – so I’d rather not say yes put me out now – give me some time – any amount of time to get that affair in order" (ID10)* 3. *“Maybe so I or my friends or family could say goodbye to me – because it does very much sound like the end of life and I think I would prefer that level of closure for me or other people than to just pass away immediately” (ID13)* 4. *"if you know you have 3 weeks to live there are a lot of things that maybe you might like to put your life in order but if you pass away its – you know – you don’t get to say goodbye" (ID11)* 5. *“but just to let my family know that I am here and they can say their goodbyes" … "Dying immediately is horrible – and the impact on people – say for my daughter” (ID9)* |
| **Valuing time in the future** | Valuing time in the future different due to expected health state | 1. *“but you have to look at the overall situation as to what problems are going to appear in that time … you can’t predict that – you don’t know if you are going to break your leg or get into Alzheimer’s or something like that – so you live for 15 years – I would stick to option B because 18 years its neither here nor there - having worked with a lot of elderly people in 18 years’ time I could be bed bound with a broken hip” (ID9)* 2. *“I guess are you talking about me – my age I’m 62 so now 77 – I’m staring down the barrel of 90. I picture – what sort of life am I living at the end of it” (ID10)* |
|  | Anticipating different preferences for the elderly | 1. *“Probably people at different age group would have different opinion” (ID7)* 2. *"I guess it depends on what age a person is when you are asking this question to them – if they are 90 2 years is not going to make a lot of difference to them – if it’s a younger person they might want to live for an extra 2 years" (ID12)* 3. *“I guess it would depend on how old you were when you were doing this. If you were in your 70s/80s maybe you’d take option B but if you were a lot longer … again this question would depend on the age you are – if you are in your 80s having 4 great years with really no issues it isn’t such a bad thing. If you were in your 50s or 60s then I’d put up with some moderate pain and slight problems washing myself” (ID19)* |
|  | Differences between 8 and 10 years, 15 and 18 years not important | 1. *“If you were in your later years and you had something wrong with you then 8 years isn’t much different to 10” (ID19)* 2. *[15 vs 18] “they are virtually the same” (ID15)* 3. *“the duration is insignificant to me – if I’m going to go with 8 years of moderate pain vs 10 years of slight pain – extreme anxiety to me" "3 years is no biggy – I’d rather have 15 years that are good than an extra 3 that are not going to be good enough" (ID10)* 4. *“To be honest, the duration didn’t matter. Unless you give me the next slide that says 5 years or 2 years…… I can’t see 8 years and 10 years being that much difference. For me, if I think extreme anxiety or depression, I think about someone sitting at home, they can’t go out and do anything, they have a very poor quality of life, why have an extra 2 years of that? … See 18 and 15, it's not much difference." (ID8)* 5. *“In terms of 10 years versus 8 years it's not much of a time difference I suppose for me – I mean if it was maybe like a 10 versus a 5 then I might maybe take the lifespan into my consideration but because it's only 2 years it feels like it’s [the health loss] not really comparable. (ID2)* 6. *“Straight away I see 15 18 years is 3 years difference to me so in my mind I’m eliminating that – 3 years doesn't seem much so I’m now looking down the rest of them. I can knock out mobility they are the same so and knock out pain they are the same so I’m just comparing the other differences there." … “If it was 20 yrs versus 15 I probably would have gone with option A” (ID1)* |
|  | Treating time differences as absolute value | 1. *[15 vs 18] or [2 vs 5] “the difference is still the same” (ID20)* |
|  | Treating time difference as potentially constant proportional trade off (for some decisions but not all) or discounting differences in duration that occur in the future | 1. *[15 vs 18 years] "difference in years isn’t that great"; [10 vs 7 years] “that is still long enough that it probably wouldn’t influence me that much; [5 vs 2 years] “that would definitely make me reconsider or change my thinking about it” (ID13)* 2. *“I am leaning towards A, cos it is such a big difference between 3 and 2 years. It's not 1 year where if you said, 10 and 11, to me that’s no big deal. It’s when it's 3 and 2 – that is a big deal." "If I was to be analytical here, if I was to compare them and say 9 is like 60% of 15, I think (off the top of my head), so let's just say, when it's like… that’s a big difference. When it’s like 2/3 of it, or when you’re talking like less than a quarter or something, like if it was say 15 and 12, 12 to 15, that’s only a little bit less. That’s like less than a quarter less." "Yeah, see I think I would have to go with C. I just picked it out straight away. To have one where you have got no issues at all and 6 months to a year, I don’t look at it as half the time – I look at it as 6 months difference. That sounds silly, but I am not looking at it like that… I am looking at it like its’ only 6 months difference, but in 6 months imagine being able to do everything you want, do everything for yourself, you’ve got no pain, you’re not anxious ... you’re basically perfect." (ID8)* |

**Theme: Process of decision making**

| **Sub-theme** | **Description** | **Example quotes (#)** |
| --- | --- | --- |
| **Process for interpreting the health state or the choice offered** | Drawing on own experience (self or others) of the state | 1. *“I’m just thinking the worst pain I’ve had – childbirth – rolling around for 3 days in that kind of pain – I can’t imagine doing that for years" (ID1)* 2. *“Yeah, and I do think some of these things you can work around. So, it may not be as bad as they seem. I’ve had knee problems for 10/15 years, I am used to pain. (ID8)* 3. *“to some degree I was also drawing on what I had to go through with mum” ... "moderate pain would be a constant – I guess I’m drawing on my own experience – I had a disc protrusion – and for a while there it was really getting ugly – I was living with it for a while and thinking this was bearable but it got to a point where I was double over on a chair saying to a colleague I can’t live like that – so moderate pain is me sitting there bearable – extreme pain is me sitting over the chair trying to relieve the pressure – to get away from the pain " (ID10)* 4. *“...because I know people with severe depression and close family members, and I know what they are going through and it's not good" (ID12)* |
|  | Hard to value health attributes in which experience is lacking | 1. *“I think maybe having been in a similar situation myself it kind of makes it easier to imagine that type of scenario. If I hadn’t felt anxious or anything that is similar to that I don’t think I’d be able to relate to that situation” “If it were to be saying mobility wise – because I haven’t experienced that I might not perhaps give it as much weight even if it would be purple or pink” … “think having experienced some of pain and anxiety that helped a lot whereas if I’m looking at say relatives experiencing that I mean I might physically see you know - see the difficulties that they might have but aside from that item I don’t think I can fully relate to it as much” (ID2)* 2. *“It may be hypothetical, but you are actually relating to your own personal experience and I think it's difficult to sort of imagine a hypothetical scenario when you are constantly thinking that may or may not relate to me (ID9)* 3. *“I’m trying to draw on past experience of when I was that depressed or upset but I can’t come up with a reasonable scenario that I can draw on to be honest – maybe in personal life with a break up of a relationship or something like that but even then I was never in a scenario that I wanted to kill myself”...”I guess everyone has been immobile at some point” (ID10)* 4. *“I don’t know what it would be like to not be able to jump in the shower and have a wash – so they are hard to imagine" "I don’t want to be extremely anxious, and I don’t want to be moderately anxious – I am neither of those in normal life. So hypothetically – how do I – it's very hard for me to ascertain what an extremely anxious person is going to feel like – as to oppose to what does moderately mean – if I don’t have slight anxiety, I don’t know what slight anxiety will mean" (ID5)* 5. *“I don’t know, it's hard cos I don’t know what moderate depression and anxiety is like.” (ID8)* 6. *"Yes because I have never had any of those conditions so it is hard to imagine what it would be like" "I have personally never experienced any mental illness so trying to qualify that against physical pain was quite difficult" (ID21)* |
|  | Some states judged to be unrealistic | 1. *“For B the anxiety might as well be there as well because I don’t think you can have all that unable to walk and self-care and not be depressed about things” (ID2)* 2. *“There were some when it was extreme pain and you can’t do your usual activities but I could walk around fine – something like that is difficult to imagine – didn’t quite seem realistic – walk around fine but I can’t dress myself.” (ID1)* |
|  | Hard to value unrealistic states | 1. *"although if you are in severe pain you are not going to have a time when you are completely able to do your usual activities anyway so really" [referring to extreme pain and no problems with usual activities] "that's not a health state" "so that's a junk question" "because if you are in severe and extreme pain there is no way you are going to have no problem doing all of your usual activities – I can’t think of any medical condition where that would be true" "I just think that the extreme pain and totally being able to do usual activities is the only one that is really truly not possible" "the reason I can’t imagine the one is that you literally can’t have that health state” "the first one so that’s not actually really … that doesn’t make any sense - that space doesn’t exist." (ID3)* 2. *“I would doubt that if you had all of these ‘unables’ in B you wouldn’t be suffering from A&D – so it's sort of falsifying what column B would actually be." (ID5)* |
|  | Lack of confidence in their decision as missing details on medication/treatment | 1. *”that’s a hard one – can I ask in choosing this option – especially the ones having some difficulties in pain, discomfort and so on – is the question of being medicated – or am I living without medication” (ID2)* 2. *”When you say severe pain – are you talking without any medication to deal with the pain? (ID10)* 3. *“Is this how I feel even given medication or is this before any mediation is given?” (ID13)* |
|  | Lack of confidence due to uncertainty in ‘usual activities’ | 1. *” I feel like it would be maybe clearer if instead of usual activities it kind of had specific issues like you have no hearing or eyesight or cognitively impaired – just usual activities does make it a little confusing – if I can’t do this what is my actual state. (ID13)* |
|  | Lack of confidence in their decision as it is only hypothetical | 1. *“Not really – look if I was in the situation, it might be harder” (ID3)* 2. *"It's hard without being in that situation to make these judgments. I’m trying to put myself in that situation” "I mean it is hard because I’m not in that situation" "if I was actually in that situation I might choose differently – who knows" (ID1)“That’s a difficult decision – and I may swing - if you ask me tomorrow I might swing the other way” (ID6)* |
|  | Impact of a health attribute varies depending upon the level of other health attributes | 1. *"even if the person has some physical disabilities moderate depression would allow to cure this person or help this person easier" …"– it will be easier to interact with such a person therefore it will be easier to help such a person"... " and in curing physical pain it’s very important that people are positive – that actually helps many people to have a stronger immune system if they are positive – to be more resilient and to cope with – with an unfortunate situation (ID7)* 2. *[low anxiety and depression would mean you can] “work on yourself to overcome problems if you choose to” (ID4)* |
|  | | |
| **Interactions between duration and health state: Multiplicative or additive thought process** | Multiplicative approach: Considering health attributes in combination with the duration of the health impairment | 1. *“But I went with A mostly due to the pain. And I don’t know if it’s a good thing to live 2 years longer or if it’s a bad thing if everything was extreme then I’d probably go for the shorter life. I look at them all separately and then look at it overall” “and weight up the pros and cons” (ID1)* 2. *“For 6 more years I can get a lot more done and do a lot more and have more life – and there is nothing show stopping that I can’t do anything – or that it’s too painful to get out of bed – or I’m too anxious and depressed to leave the house – so there is still a good amount of life I can actually live” (ID3)* 3. *“I mean it’s a lot of extra years to put up with a considerable number of extra problems – I find life valuable – I’d probably go with B because of the number of extra years – even though it’s quite demanding in terms of what I need to put up with” (ID6)* |
|  | Additive approach: considering health attributes and the duration attribute separately without consideration of their interaction | 1. *“I went for the colours first because that kind of told me the severity of the situation and then I looked to what item it is related to and then I worked my way down to the less severe for each scenario – and then figured out whether it was worth the duration of life span.” (ID2)* |
|  | Additive approach: ignoring health attributes that are similar when durations differ | 1. *"..for me I think I just put a line through it because – you have similar problems – what you need to think about is what is the difference between the 2 lives and why would you prefer 1.” (ID11)* 2. *“I basically eliminate them if they are the same – and I look at other parameters” (ID7)* |

**Theme: Survey design**

| **Sub-theme** | **Description** | **Example quotes (#)** |
| --- | --- | --- |
| **Wording around the ‘dead state’: Passing away vs dying immediately vs not living any time** | ‘Passing away’ gentler, softer, kinder, more polite than dying | 1. *“passing away has a much softer tone to it” (ID2)* 2. *“passing away is gentler” (ID4)* 3. *“I prefer this phrase [ref to passed away] – definitely – when you say dying it makes me feel some negative atmosphere – I don’t feel good with it" (ID7)* 4. *“it sounds somewhat better than dying – it takes that edge of it a little bit” (ID18)* 5. *“it’s a softer way of saying before you die – it’s a gentler way of saying it – than hitting someone over the head and saying you are going to drop dead” (ID10)* 6. *“I will say he has passed away because it seems a more polite way of saying it – and if you say he has ‘died’ people are more shocked” (ID9)* |
|  | ‘Passing away’ infers a different type of death to dying | 1. *“if I see it’s before death I think you are getting killed or something – it seems very sudden – but passing away makes it seem like it could be anything” (ID13)* 2. *“passing away is passive” “[dying] is not a very nice word to use…it is aggressive” (ID15)* |
|  | Happy with any language | 1. *“personally it doesn’t bother me at all” (ID8)* |
|  | Preferred the term ‘dying’ | 1. *“I’m sometimes pretty honest and blunt so I’d probably say before dying” (ID21)* |
|  | The term ‘dying’ easier for participants with ESL | 1. *“You can say whichever you want – but if you are going easy English I would say dying – it’s very clear. ...dying is much clearer – if somebody doesn’t understand a language well using a euphemism may confuse them if they really have very low language literacy” (ID3)* |
|  | Emotive response from participants due to confronting language | 1. *“Dying immediately is horrible because people say I knew [name] yesterday and last night he died – and the impact on people … but to immediately pass away is too emotive – its oh my god you’ve passed” (ID9)* 2. *“It is very clear but it brings some negative feeling to me – death. The word dead someone is cold it makes you feel cold and uncomfortable” (ID7)* |
|  | Participants disliked the phrase ‘not living any time at all’ | 1. *“That one is hard to read - it’s like you are about to be put in the electric chair or something – like you have done something wrong.” (ID1)* 2. *“not live any more time at all – that is not nice – it's rude and arrogant.” (ID15)* 3. *“I think – not live any further time at all doesn’t sound very right” (ID11)* 4. *“not live any further time at all that’s quite a cold statement and I would not appreciate anyone saying that” (ID9)* |
|  | Recommendations for other terms | 1. *“Before your life ends – I think that is more neutral” (ID7)* |
|  | Interpretation of ‘dying immediately’ | 1. *Option C [dying immediately] wouldn’t be so bad either because I wouldn’t know any different (ID1)* 2. *“It says your times up – could be a heart attack or a car accident – could be a lot of things but it's sudden. It basically mean your time is up” (ID6)* 3. *“Just immediately pass away to me is not, you’d better finish up everything cos at 9 o'clock you’re finished. I think it’s more like, sudden. A sudden thing, like a heart attack I guess is a good example of a sudden thing”. (ID8)* 4. *For immediate death the participant thought that you are not going to die straight away anyway as it takes quite a long time once the Drs say you are dying (ID15)* 5. *“But you wouldn’t actually pass away immediately – it's not like we are just going to pull the plug on your in 5 minutes if you chose the ‘pass away immediately’ – “Its just saying its palliative care – that’s what I’m guess that’s what you are actually trying to say”... "I’d just think stop treatment and let me pass away naturally from what it is and don’t prolong life because I think that’s actually what you are trying to ask" (ID3)* |
|  |  |  |
| **Wording around the ‘dead state’: Example of heart attack** | Heart attack example easy to understand and acceptable | 1. *“everyone understands it” (ID10)* 2. *“It’s very clear – very easy to understand … I think this is totally acceptable” (ID7)* 3. *“you are going to say that someone passed away suddenly – you are probably either going to be something like heart attack – it's probably what people think about – that or car accident – but if you are talking about health then that is probably most appropriate” (ID20)* 4. *“everyone knows that just an easy obvious way to say that’s how they died instead of hit by a bus or something” (ID1)* 5. *“it’s a real scenario” (ID2)* |
|  | Heart attack example harsh/scary | 1. *“scary” (ID15)* 2. *“I think the heart attack one makes it a bit too harsh” (ID6)* |
|  | Heart attack example gave additional meaning to the profile ending in heart attack and the alternative profile | 1. *“If you pass away due to a heart attack sometimes it's easier for the person concerned – but for the family that is always quite a shock – so what can I do with that” (ID9)* 2. *“It’s what they say is a good death – you just go, you’re not lingering on” (ID16)* 3. *“a heart attack gives you comfort that, OK she died of a heart attack” ... "You’ve got to I guess link it to any age and you wouldn’t say ah a 40 year old mum passed away peacefully in her sleep – like really? - whereas a heart attack can happen to anyone – it can happen to an athlete it can happen to someone who is morbidly obese – it’s a very broad term they died of a heart attack” (ID5)* |
|  | Better to have same example in both profiles | 1. *“That way they’re easy to compare. So the only difference between them is the duration of time rather than the mode.” (ID8)* 2. *“leant a bit towards B because it doesn’t confront you with how you die. Heart attacks – I’ve never had one but – people I have known who have had them they are not pleasant so – that also lends a little bit to B… if you are going to have an example in one you need to have an example in the other” (ID21)* 3. *“It makes the absence of it seem more threatening”… "If you have that [i.e. heart attack example] it implies that there could be something like a more violent or extreme death on the other one on the other side" (ID13)* |
|  | ‘Pass away’ or ‘pass away peacefully’ lacks realism | 1. *“I think the heart attack one – I think it’s a good example – because ‘passing away’ you know like peacefully or in your sleep – its kind of add a fluff to it – I suppose – kind of add the more fairy tale to it” (ID2)* 2. *[pass away peacefully in your sleep] “that’s just being a bit namby pamby” [ID19]* 3. "When yo*u say pass away peacefully in your sleep I’m only my 30s so there must have been something wrong for me to pass away in myself so that makes your table seem unrealistic if I am living fit and healthy and didn’t have any of those problems" (ID1)* 4. *“You’ve got to I guess link it to any age and you wouldn’t say ah a 40 year old mum passed away peacefully in her sleep – like really?” (ID5)* |
|  | Ethical concern for those who may have recently experienced a heart attack (Q# | 1. *“that’s a pretty common way of going – the only way you could soften it is ‘due to ill health’ – the only reason you’d say that is if you were the person filling it out and you had recently had a heart attack – that might sort of freak them out a little bit – ill health can be anything really” (ID20)* |
|  |  |  |
| **Use of colour and bolding of text** | Participants liked the use of colour | 1. *"the presentation is more interesting – and the clarity is higher – that’s beautiful” (ID7)* |
|  | Colours make decision making easier | 1. *“The colours were good because I’m getting used to seeing the deeper the colour the worst the problem” (ID1)* 2. *"having the colour would give me a quicker analysis on that – so I think that is really handy to actually have” (ID2)* 3. *“the colour coding is very helpful because instantly you can spot the differences" (ID7)* 4. *"I think the colour coding is nice because it makes it easier for me to draw my attention to the differences" (ID10)* 5. *“actually it’s quite fast for you to really look what is moderate what is slight and what is extreme" (ID11)* 6. *"Obvious when levels are the same and different – I find that very useful" …"When I look at it I’m immediately drawn to the colours in terms of comparison and I don’t even register bold or black as an indicator of differences" (ID13)* 7. *"you are drawn to them straight away – I was drawn to the deeper colours – and the wording in a bolder print – that all makes sense" (ID19)* 8. *“The colours sort of assist as well – align what is medium, low and high as well” (ID20)* |
|  | Heavy reliance on colour | 1. *"I sort of go for the option of moderate so the lighter colours rather than the darker colours." (ID5)* 2. *"Designating the colours to them makes it a lot clearer – so all I’m focusing on is the darker pink – rather than anything else"... "Easy decision because there were more pinks on option B" (ID2)* 3. *"I basically looked at the colour differences – I was really drawn to the colour differences – the first 3 items are all the same I don’t need to read that – I can see the colours are the same – you don’t need to read the whole thing" (ID10)* |
|  | Indifference to colours | 1. *"basically just the descriptions is what I used." (ID16)* 2. *“well they don’t do anything for me or not for me – they are just colours – they don’t make anything stand out – they both look pretty bland to me" (ID18)* |
|  | Meaning of colours unclear | 1. *"I don’t know why you have changed the colour of that – what the reason is behind that" (ID9)* 2. *“but I find it a distraction. I think it's because there wasn’t a key provided at the beginning so you are trying to work it – it doesn’t’ take long to work out but in some ways it’s a distraction" (ID21)* |
|  |  |  |
| **Potential** **errors and inconsistencies** | Overlooked the difference in years in choice task | 1. *“Obvious when levels are the same and different – I find that very useful – but the years I didn’t really notice that" (ID13)* 2. *“Ah – I missed that bit – so it's 10 years compared to 8 years – I guess I must have read that too quickly" (ID17)* 3. *“I think if there was a heading like the other 5 on the left I probably would have focused on it a bit more because it didn’t have a heading I probably didn’t think it was a criteria” (ID21)* |
|  | Selected choice closest to own health | 1. *“but then it says I’m extremely anxious or depressed but I’m not so or I am moderately anxious or depressed but I’m not – so what would I put – B being the closest one?” (ID9)* 2. This was also observed in ID4 where technical issues resulted in problems with the recording of the interview. |
|  | Tried to select the greyed-out choice | 1. *“Do I get the choice of 3 or just the choice of 2?” (ID16)* |
|  | Confused by the option ‘being dead’ | 1. *“What do you mean by ‘being dead? … “there’s no time frame here – that’s really difficult” (ID17)* |
|  | Health state is fluid during discussion | 1. *“But moderate would be hard to live with I imagine so I think I’ll go the shorter years with no problems. I’m just thinking of someone I know who is dying of cancer and they know they are going to die anyway and they probably want to die with no pain, no trouble, over living longer with [pause] getting about their usual activities pain and being very depressed and anxious” (ID1)* |
|  | Health state is not considered constant | 1. *"...you know the depression is one thing and I can do stuff maybe to help with that and to talk with people" (ID5)* 2. *“if I had moderate I might be able to get some help – some psychology or medication I guess” (ID16)* 3. *“and I’d probably just hope that I would have support in doing those activities – and I’ve ongoing treatment for that extreme pain and discomfort." (ID20)* |
|  | Inconsistent choices arising due to change in focus | 1. *[3 years in 55555 preferred to 3 months] “You’d go well if I can hack it out for 3 months then I’m sure I could hack it out for 3 years – so I’d go 3 years and again you are with your family for that duration of time” AND [immediately pass away preferred to 6 months in 15543] “Again I’d go the heart attack – all done” (ID5)* |

1. On four occasions technical difficulties were experienced with the Zoom platform resulting in four interviews without cameras turned on, and two interviews (ID4 and ID14) without audio recordings which relied upon note taking. [↑](#footnote-ref-1)
